# Associations Between B Vitamin Interactions with Polyunsaturated Fatty Acids and Cognitive Function Among Cognitively Healthy Older People as Modified by Amyloid Status and Sex

**DOI:** 10.3390/nu17091407

**Published:** 2025-04-23

**Authors:** Chuliang Zhao, Karen A. Abbott, Chinedu Udeh-Momoh, Geraint Price, Oliver J. K. Robinson, Sujin Kang, Celeste A. de Jager Loots

**Affiliations:** 1 AGEing Epidemiology Unit, School of Public Health, Imperial College London, London W6 8RP, UK; chuliang.zhao23@imperial.ac.uk (C.Z.); k.abbott@imperial.ac.uk (K.A.A.); chinedu.momoh@aku.edu (C.U.-M.); g.price@imperial.ac.uk (G.P.); o.robinson@imperial.ac.uk (O.J.K.R.); sujin.kang@imperial.ac.uk (S.K.); 2 School of Public Health Sciences, Wake Forest University School of Medicine, Winston-Salem, NC 27101, USA; 3 Brain and Mind Institute, Aga Khan University, Nairobi P.O. Box 30270-00100, Kenya; 4 Division of Clinical Geriatrics, Center for Alzheimer Research, Karolinska Institutet, 171 77 Stockholm, Sweden

**Keywords:** cognitive health, vitamin B12, folate, amyloid status, polyunsaturated fatty acids, nutrient interactions

## Abstract

**Background/Objectives**: Nutrients such as vitamin B12, folate (B9), and polyunsaturated fatty acids (PUFAs) may independently influence cognitive health, but their combined effects and interactions remain unclear. This study aims to investigate the effects of B12, folate, and PUFAs, including their interactions, on cognitive function in cognitively healthy older adults, considering effect modification by sex and amyloid-beta status. **Methods**: A cross-sectional analysis was conducted using data from 321 participants aged 60–85 y enrolled in the UK CHARIOT–PRO SubStudy. Dietary intake was assessed using the Scottish Collaborative Group Food Frequency Questionnaire, and cognitive performance was measured via the Repeatable Battery for the Assessment of Neuropsychological Status (RBANS). Stratified multivariate linear regression models by sex and amyloid-beta status and models with the saturated to unsaturated fatty acid ratio (substituted for PUFAs) as one of the predictors were constructed. **Results**: Males had lower total RBANS scores with a higher PUFA intake (β = −13.97, *p* = 0.04) but improved scores with increased folate (β = 9.08, *p* = 0.04). PUFA × folate revealed contrasting effects to PUFAs alone, with higher cognitive scores in the amyloid-negative group for total RBANS (β = 13.27, 95%: 3.81~22.73, *p* = 0.01) but lowered scores in the amyloid-positive group. Considering the UFA:SFA ratio, higher delayed memory scores were associated with the combined intake of folate, B12, and PUFAs (β = 7.46, *p* = 0.02) among females. In the amyloid-positive group, the negative cognitive effects observed with PUFAs were reversed when UFA:SFA was considered. **Conclusions**: Amyloid status and sex significantly influenced the cognitive effects of nutrient intake, with distinct patterns based on specific cognitive domains and nutrient interactions.

## 1. Introduction

Cognitive decline in older adults due to Alzheimer’s disease (AD) and dementia is a pressing global issue. The global incidence of dementia is expected to surge from 50 million in 2020 to 150 million by 2050 [1], while in the UK alone, the number is projected to increase from 850,000 individuals currently to 1,350,000 by 2040 [2]. Moreover, dementia incurs profound economic and social costs, affecting healthcare resources and family support structures [3]. Numerous lifestyle factors, including low education and physical activity levels, cardiovascular health, malnutrition, depression, social engagement, brain injuries, hearing loss, and poor eyesight are known to influence dementia risk [4,5,6]. The preclinical stage of AD, before the first clinical symptoms become apparent, may be characterized by mild cognitive impairment but no dementia. This stage is thought to offer the optimal chance of therapeutic benefit, as the underlying neuropathology may still be reversible [7].

Emerging evidence also points to the critical role of certain micronutrients, especially B vitamins and omega-3 fatty acids, in cognitive health [8]. B vitamins (B6, B12, and folate) help maintain the optimal level of homocysteine (Hcy) through the methionine cycle [9,10,11]. Elevated Hcy levels and low levels of B vitamins can impair blood flow and contribute to neurodegeneration, leading to cognitive decline [12,13]. Moreover, evidence shows that B-vitamin supplementation is effective in reducing Hcy levels [14], with benefits observed in episodic and semantic memory and global cognition [15], especially when applied at an early stage [13], among those with early or mild cognitive impairment (MCI) with elevated Hcy levels [14,15] and with long-term interventions [16]. Interestingly, higher B-vitamin levels might not always yield better cognitive outcomes—some studies suggest that high folate or B12 levels correlate with accelerated cognitive decline [17] under specific circumstances, such as in individuals with certain genetic polymorphisms [18] and with a high intake of folate exceeding 400 μg/d [19].

**Figure 1:**
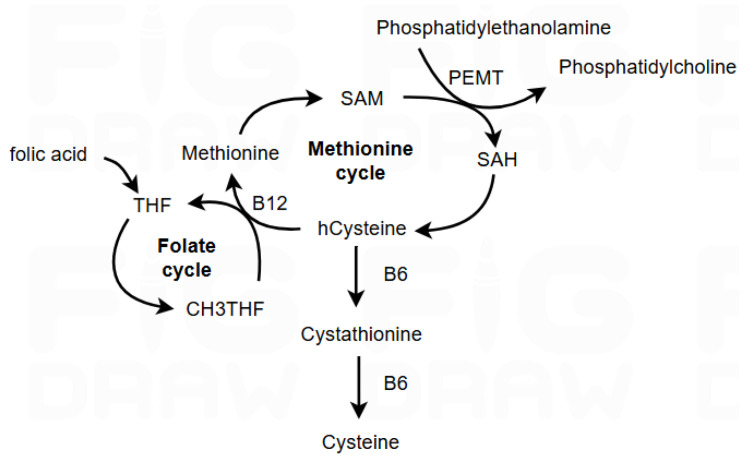
Metabolic interactions in homocysteine methylation, the folate cycle, and phosphatidylcholine synthesis via the PEMT pathway. Figure 1 was created using FigDraw.com.

Furthermore, the interaction between nutrients plays a role in the association between B vitamins and cognitive performance [11,20,21,22,23]. Van Soest et al. [8] used a nutritional index (Hcy, omega-3 folic acid + vitamin D status) and showed a four-fold increased risk of dementia in those with the most suboptimal nutritional index, which is higher than the risk from diabetes and presence of the APOEε4 allele. Research highlights that combined supplementation of B vitamins (B6, B12, and folate) has a more substantial effect on lowering Hcy and inflammatory markers than single nutrient interventions, especially in those with MCI [8,12,20,24]. Additionally, omega-3 fatty acid (*n*-3 FA) status, especially the docosahexaenoic acid (DHA) component, may enhance the protective effects of B vitamins on cognitive function by supporting cell membrane integrity and reducing Hcy [25]. Phosphatidylethanolamine (PE) is converted into *n*-3 FA-enriched phosphatidylcholine (PC) via the phosphatidylethalonamine *N*-methyltransferase (PEMT) pathway, mainly in the liver and when Hcy levels are low, due to the B-vitamin (B6, B9, B12) regulation of methylation (Figure 1) *n*-3-FA-enriched PC is made available via plasma to the brain and other tissues, ensuring phospholipid balance, good neurotransmission, and reduced brain atrophy, thus contributing to cognitive health [25].

Nutrient interactions affecting cognition may be influenced by various confounders and modifiers. One such example might be high levels of amyloid beta (Aβ) in the brain [21], which is one of the neuropathological hallmarks of AD as applied to the amyloid, tau, and neurodegeneration [AT(N)] biological construct to classify AD [26]. This classification scheme is also used for staging the severity of cognitive decline; thus, the greater the burden of amyloid and tau is, the greater the risk of cognitive decline [27]. Amyloid deposition in asymptomatic older adults has been associated with brain atrophy but not with symptomatic AD [28]. However, the relationships between brain Aβ status and nutrient intake have only been sparsely investigated [29]. A higher intake of vitamin B12, vitamin D, and *n*-3 PUFAs was associated with a lower cerebral Aβ burden in a small number of cross-sectional studies using PET imaging reviewed by Hill et al. [30], while low levels of B vitamins and PUFAs have been associated with increased brain atrophy [31,32]. Recent studies have implicated an independent linkage of elevated Hcy levels with amyloid accumulation [28], and low Hcy levels facilitate the protective effect of PUFAs against atrophy [23]. RCTs with both DHA and folic acid supplementation have shown decreases in CSF tau and plasma Aβ, respectively [33]. Diet-induced elevated Hcy has been shown to reduce amyloid-beta clearance from the brain, while folic acid supplementation in mice reduced Aβ oligomer-induced neuronal toxicity [34]. Additionally, the chronic oxidative stress from amyloid accumulation may accelerate PUFA peroxidation, exacerbating neuronal damage and contributing to nutrient imbalances [34].

A limitation in the existing literature is that most studies only focus on the effect of B vitamins on global cognition, while associations of nutrients with specific cognitive domains are still unclear [35]. We have previously shown that sex is a modifier of responses to B-vitamin status on attention and immediate memory, especially in those with the most deficient blood levels of B vitamins [9]. However, this study is the first to determine the differing effects of nutrient intake as related to the preclinical levels of the AD marker Aβ deposition. This study seeks to address gaps in the literature by examining the associations between the interaction of B vitamins (B9 and B12) and PUFAs with cognitive function in cognitively healthy older adults in our cohort. It is a cross-sectional study designed before conducting a longitudinal study to explore the long-term effects of nutrient intake on cognitive function. Specifically, we aimed to assess how single nutrients and nutrient interactions relate to cognitive subdomains and to explore potential modifiers of the effects (viz., sex and amyloid status). The findings could help inform nutritional guidelines and interventions targeting cognitive decline in aging populations.

## 2. Materials and Methods

### 2.1. Study Population

**Figure 2:**
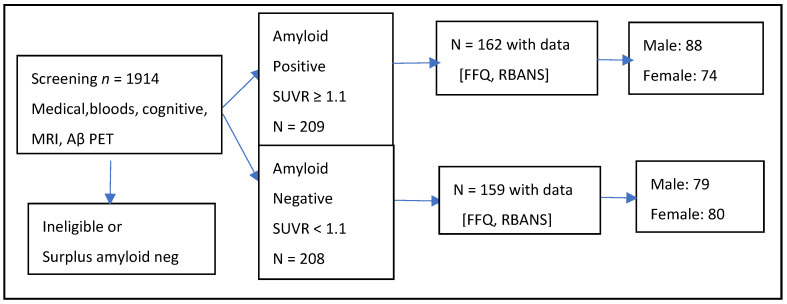
Study design flowchart depicting the participant numbers included in the analyses.

Participants for this cross-sectional sub study were recruited from the Cognitive Health in Ageing Register: Investigational Observational and Trial Studies (CHARIOT) in Dementia Research: Prospective Readiness Cohort SubStudy, initiated by Imperial College London in 2015, and the recruitment was assisted by primary care practices in Greater London. A total of 1914 individuals, aged 60 to 85 years, were screened, out of which 417 participants were successfully enrolled (Figure 2). The exclusion and inclusion criteria are detailed in the CHARIOT SubStudy protocol [36].

The eligible participants underwent a comprehensive evaluation process, which included a medical history assessment, a physical examination, and laboratory tests. Baseline data collected included demographic information (age, sex, educational level), height, weight, and blood pressure. Blood samples were taken to measure various biochemical markers, such as high-density lipoprotein (HDL), low-density lipoprotein (LDL), creatinine, triglycerides, and blood levels of vitamin B12 and folate. If participants had vitamin B12 blood levels below 148 pmol/L, additional plasma tests were performed to confirm the deficiency [36].

### 2.2. Amyloid-Beta Measurement

An equal number of amyloid positive to negative participants were enrolled into the CHARIOT–PRO SubStudy to enable comparisons of risk factors for cognitive decline at the preclinical AD stage. Amyloid status was measured by amyloid positron emission tomography (PET) using F18-radiolabeled amyloid tracers. Standardized uptake value ratios (SUVRs) were formed by normalizing the composite multiregional target regions of interest (ROIs) to the cerebellar crus gray matter [37]. The amyloid PET target meta-ROIs included the prefrontal, orbitofrontal, parietal, temporal, anterior, and posterior cingulate and the precuneus. The threshold for amyloid positivity used was SUVR = 1.1. For those participants not receiving Aβ PET, CSF samples were collected during screening and analyzed for AD-related markers including amyloid beta. The Aβ data were used for the determination of enrolment eligibility [36].

### 2.3. Nutritional Assessment

The nutritional assessment in the study was conducted using the Scottish Collaborative Group Food Frequency Questionnaire (SCG FFQ) [38]. This questionnaire was voluntarily self-completed by participants to capture their habitual dietary intake. Participants were asked to report the frequency and portion size of their consumption of various food items, including fruits, vegetables, dairy products, meats, fish, cereals, snacks, and beverages. The data collected from the SCG SFFQ were then computed to estimate the intake of specific nutrients, such as vitamins, minerals, and macronutrients. In particular, for this study, folate, vitamin B12, and PUFA intake and the unsaturated fatty acid (UFA)/saturated fatty acid (SFA) intake ratio were utilized. The UFA:SFA ratio was calculated as mono-unsaturated fatty acids (MUFAs) + PUFAs/SFAs.

### 2.4. Cognitive Tests

The Repeatable Battery for the Assessment of Neuropsychological Status (RBANS) is a 20 min cognitive assessment made up of 12 subtests, which evaluate five cognitive domains: “Attention (Digit Span and Coding subtests), Language (Picture Naming and Semantic Fluency), Visuospatial Construction (Figure Copy and Line Orientation), Immediate Memory (List Learning and Story Memory), and Delayed Memory (List Recall, List Recognition, Story Recall, and Figure Recall subtests)” [39]. The combined scores from these domains are converted into a total scale score adjusted for age, represented as a t-score with a mean of 100, a standard deviation (SD) of 15, and a range of 40–160. The effectiveness of RBANS has been verified through its correlation with daily functional abilities in patients with MCI and with the clinical dementia rating in both MCI and AD [39]. In this study, the RBANS was conducted with participants by trained assistant psychologists.

### 2.5. Statistical Analysis

Among the 417 participants enrolled, data were only available for the 340 who completed the SCG SFFQ. Nineteen participants with missing values in RBANS total scores, BMI (calculated by weight/height^2^), and education level were excluded, as were those with outlying serum vitamin B12 (>600 pmol/L) and folate (>100 nmol/L). Nearly 30% of participants had missing data in one or more RBANS index score calculations; therefore, multiple imputations were performed using the MICE package, with predictive mean matching to handle missing data [40]. After exclusion, 321 participants remained for analysis. Power analysis, including the *t*-test (Cohen’s d = 0.5), correlation analysis (r = 0.3), ANOVA (f = 0.25), and chi-square test (Cohen’s w = 0.3), was conducted to ensure that the available sample size was adequate to detect meaningful effects.

RBANS scores, nutritional intake, and potential confounders (demographic variables, blood biomarkers, medical history, smoking status, and APOE status) were compared between males and females and between amyloid-positive and amyloid-negative groups using *t*-tests for normally distributed variables, the Mann–Whitney U test for skewed variables, and the chi-squared test for categorical variables. Correlation tests between RBANS scores, nutritional intake, and potential confounders were performed with Spearman’s rho, ANOVA, and the chi-squared test to determine potential confounding factors to include in the regression models.

Sex-specific and amyloid-status-specific multiple linear regression models were created with independent predictors of the interaction term between nutrient intake and PUFAs (continuous), vitamin B12 (continuous), and folate (categorical; high, medium, and low). BMI (continuous) and education level (categorical; masters/higher, bachelor, secondary school/lower) were adjusted as independent covariates. Dependent outcomes included the total RBANS and RBAN index scores by cognitive domain (immediate memory, delayed memory, attention, language, visuospatial construction). The folate intake in the model was categorized into tertiles because previous research has shown that the relationship between folate and cognitive function is not linear but rather follows a “J”-shaped curve. This suggests that both low and high levels of intake may negatively impact cognitive function [41]. Using categorical predictors allowed us to accurately assess these effects. The nutrient intakes were standardized with z-score normalization [42].

A second model was built to additionally adjust for confounders of serum biomarkers, including HDL, LDL, and triglycerides (all continuous), and medical history, including CVD and endocrine and metabolic diseases (EMs) (all categorical; yes, no). A third model was built to further adjust for smoking status (categorical; no, yes, stopped) and APOE ε4 allele carrier (categorical; yes, no). Multiple linear regression models for all participants combined were also produced, and the results are presented as Supplementary Material.

Furthermore, the UFA:SFA ratio was utilized in the models instead of PUFAs to control for the intake of SFAs, which may be associated with negative cognitive outcomes. This may highlight the benefits of unsaturated fats more clearly and account for the potential synergistic effects of different types of fats on cognition.

For sensitivity analysis, models were built with blood levels of vitamin B12 and folate as separate predictors, with the total RBANS score as the dependent outcome. Additionally, linear models using education level as a categorical predictor examined the separate intake of vitamin B12, folic acid, and PUFAs to determine whether education could influence the intake of nutrients, potentially masking the association between nutrient intake and cognitive performance.

A *p*-value less than 0.05 was considered statistically significant. The estimates (β) generated by the models were presented with 95% confidence intervals (CIs). All the data analyses were conducted in R, version 4.3.1.

### 2.6. Ethical Approval

The CHARIOT–PRO SubStudy received National Research Ethics Services approval (NRES) Committee London Central (reference 15/LO/0711 (IRAS 140764)) and internal Imperial College London Research Ethics and Joint Research Compliance Office approval. The study is registered at www.clinicaltrials.gov (NCT02114375). All participants received comprehensive information about the study’s procedures, risks, and benefits and provided their informed consent.

## 3. Results

### 3.1. Descriptive Results

The participants in the study had a mean age of 71.78 years (±5.49 years). The population was almost evenly divided by sex, with 47.98% female and 52.02% male. Approximately 40%, defined by BMI 25–29.9 kg/m^2^, were overweight, and 14.0%, defined by BMI ≥ 30 kg/m^2^, were obese in this cohort.

Females exhibited significantly higher blood levels of vitamin B12, HDL, and LDL cholesterol than males (*p* = 0.001), while males had significantly higher serum creatinine levels (*p* = 0.001) and a higher prevalence of smoking and CVD and a higher education level (*p* < 0.05). No significant sex difference in nutrient intake was detected. Females demonstrated superior performance in immediate memory and language tests (*p* = 0.001).

Detailed characteristics of the participants are presented by amyloid status in Table 1. The amyloid-positive group was older than the amyloid-negative group (*p* = 0.03). It also had more APOEε4 allele carriers (*p* = 0.001) and a higher level of serum folate (*p* = 0.02). Both the RBANS total and index scores were comparable across the two groups. The correlation test showed that only education level correlated with scores in total RBANS, attention, and immediate memory (*p* < 0.05).

**Figure 3:**
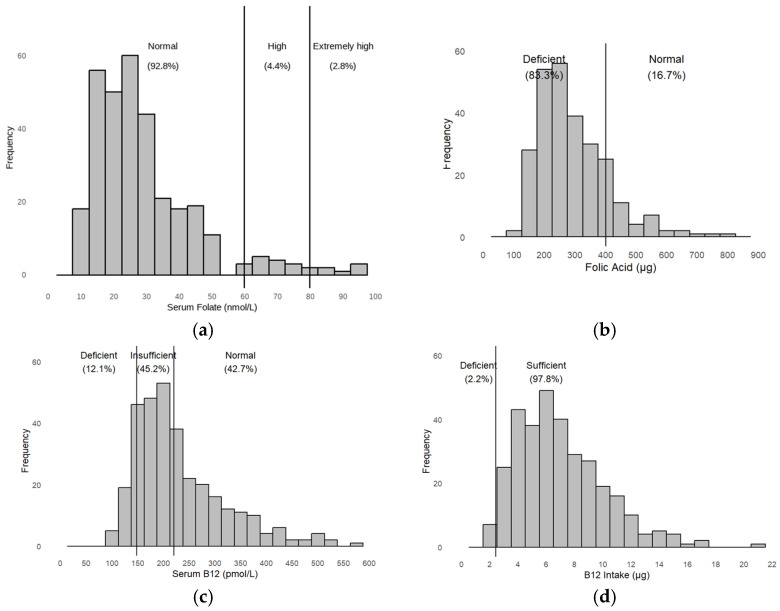
Histograms display the distribution of serum and intake levels of folate and vitamin B12 in the study population: (**a**) serum folate, (**b**) folate intake, (**c**) serum vitamin B12, (**d**) vitamin B12 intake.

The median values of serum folate and vitamin B12 were within the normal range, respectively, at 206 pmol/L (IQR = 107 pmol/L) and 25.8 nmol/L (IQR = 15.45 nmol/L) (Figure 3). Noticeably, there are discrepancies between the proportions of B-vitamin deficiency according to serum versus nutrient intake levels (Figure 3b,d).

### 3.2. Multivariate Model Results

For models with PUFAs as one of the predictors:

- **For all participants**

Sex (Model 1) and education (Models 1–3) were significantly associated with RBANS total scores (Table 2a). Only vitamin B12 intake was associated with improved performance in language (β = 8.86, 95%: 1.16~16.55, *p* = 0.02), specifically in Model 1 (Table 2b). See Supplementary Table S1 for results of all domains.

**Figure 4:**
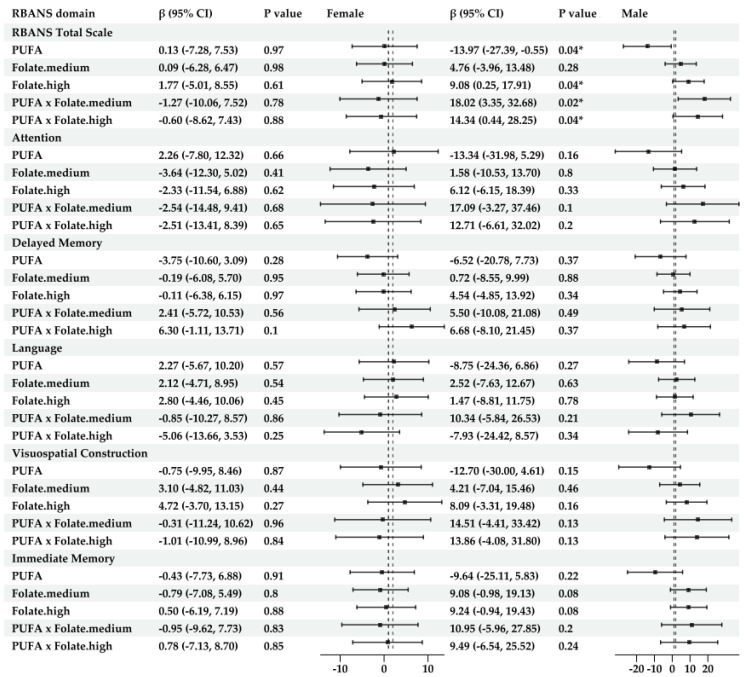
Forest plot of results for interaction terms between PUFAs, B12, and folate (FA) in the sex-stratified linear regression models for the RBANS total and index scores (Model 2 ^1^). ^1^ Model 2 was adjusted for BMI, education, sex, HDL, LDL, triglycerides, CVD history, and endocrine and metabolic disease history. The results of Model 2 are presented in this figure and later in this paper as the main results, as most of them had the lowest AIC values among the three models, indicating the highest goodness of fit. * indicates statistical significance at *p* < 0.05.

- **For sex-specific models** (Figure 4)

Significant associations were only found in males. Their scores for total RBANS were lower with the increased intake of PUFAs (β = −13.97, 95% CI: −27.39, −0.55, *p* = 0.04), but higher scores were associated with increased folate intake (β = 9.08, 95% CI; 0.21, 17.91, *p* = 0.04) and with the interaction between these two nutrients (β = 18.02, 95% CI: 3.35, 32.68, *p* = 0.02). LDL was a significant predictor of immediate memory scores in Models 2 and 3. See Supplementary Table S2.

**Figure 5:**
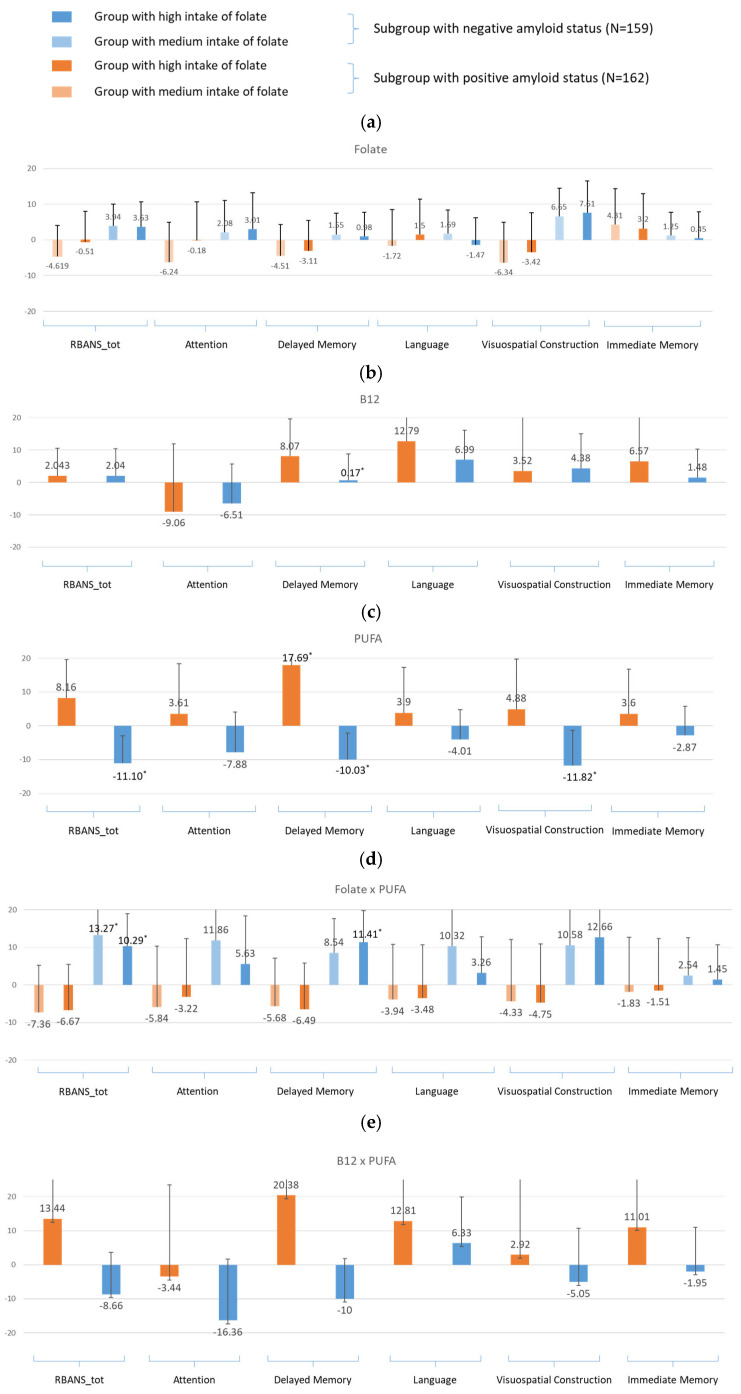
Bar graphs of the estimates for the nutritional intake in the amyloid-status-stratified linear regression models for the RBANS total and index scores (Model 2 ^1^) for (**a**) folate, (**b**) vitamin B12, (**c**) PUFAs, (**d**) folate × PUFA, (**e**) vitamin B12 × PUFA. ^1^ Model 2 was adjusted for BMI, education, sex, HDL, LDL, triglycerides, CVD history, and endocrine and metabolic disease history. * indicates statistical significance at *p* < 0.05.

- **For amyloid-specific models** (Figure 5)

In the amyloid-positive group, the intake of PUFAs was associated with a trend of higher scores for total RBANS and the subdomains and was significant for delayed memory (β = 17.96, 95% CI: 1.31, 34.61, *p* = 0.03). However, in the amyloid-negative group, the trends were negative, significantly in total RBANS (β = −11.10, 95% CI: −19.27,−2.92, *p* = 0.01), delayed memory (β = −10.03, 95% CI: −17.90, −2.15, *p* = 0.01), and visuospatial construction (β = −11.82, 95% CI: −22.25, −1.39, *p* = 0.03). With folate intake, there were no significant associations with cognitive performance, although in some domains, the trends for the amyloid-positive group were in the negative direction. B12 intake was generally associated with a trend in higher RBANS scores in both the amyloid-positive and -negative groups, except for the domain of attention (Figure 5c). See Supplementary Table S3.

The interaction effects between PUFAs and folic acid followed a similar pattern to that of folate intake alone, with cognitive scores exhibiting a negative trend in the amyloid-positive group. However, PUFA × FA was associated with significantly higher RBANS scores in the amyloid-negative group, especially for total RBANS (β = 13.27, 95% CI: 3.81, 22.73, *p* = 0.01), delayed memory (β = 11.41, 95% CI: 3.04, 19.78, *p* = 0.01), and visuospatial construction (β = 12.66, 95% CI: 1.57, 23.76, *p* = 0.03). Conversely, the synergistic effect between PUFAs and B12 displayed a similar pattern to that of PUFAs alone, showing a positive trend in the amyloid-positive group (except attention), significance for delayed memory in Model 1, while revealing a negative but non-significant trend in the amyloid-negative group. When considering the interaction effect among all three nutrients, a positive association was found for attention (β = 19.05, 95% CI: 0.14, 37.97, *p* = 0.05) in the amyloid-negative group, while a negative association was observed in delayed memory for the amyloid-positive group (β = −25.45, 95% CI: −47.41, −3.49, *p* = 0.02). The results for all models are in the Supplementary Material, Tables S1–S5.

For models with the UFA:SFA ratio substituted for PUFAs as one of the predictors:

- **For all participants**

As expected, the inclusion of SFAs in the models affected cognitive scores negatively in general. Increased UFA:SFA intake was negatively associated with scores in both the total RBANS (β = −3.75, 95% CI: −6.92, −0.57, *p* = 0.04) and in delayed memory only in Model 1 (β = −3.18, 95% CI: −6.29, −0.07, *p* = 0.045). However, in this model, B12 intake was associated with improved scores in delayed memory (β = 5.08, 95% CI: −0.08,10.24, *p* = 0.05) and language (β = 5.46, 95% CI: 0.18, 10.52, *p* = 0.034). When the synergistic effect of folate with UFA:SFA was considered, scores in language were lowered (β = −6.04, 95% CI: −11.30, −0.41, *p* = 0.02).

**Figure 6:**
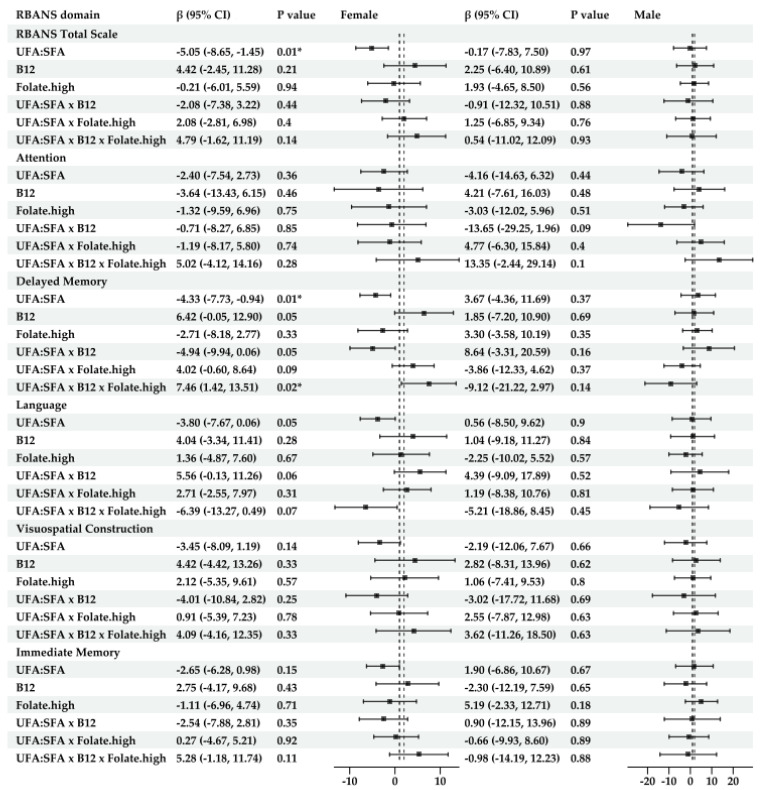
Forest plot for the sex-stratified linear regression models for RBANS total and index scores with the predictors UFA:SFA, vitamin B12, and folate and interactions (Model 2 ^1^). ^1^ Model 2 was adjusted for BMI, education, sex, HDL, LDL, triglycerides, CVD history, and endocrine and metabolic disease history. * indicates statistical significance at *p* < 0.05.

- **For sex-specific models** (Figure 6)

The analyses with UFA:SFA showed significant negative associations in females for RBANS total scores (β = −5.05, 95% CI:−8.65, −1.45, *p* = 0.006), while HDL was a significant predictor of the cognitive scores for Models 2 and 3. Female scores in delayed memory were negatively associated with the rising UFA:SFA ratio (β = −4.33, 95% CI: −7.73, −0.94, *p* = 0.01) and with the interaction between B12 and UFA:SFA (β = −5.21, 95% CI: −10.28, −0.13, *p* = 0.05) but were positively associated with the interaction between folate × B12 × UFA:SFA (β = 7.46, 95% CI: 1.42, 13.51, *p* = 0.02). Moreover, their language scores were lower with UFA:SFA alone (β = −3,97, 95% CI: −7.89, −0.04, *p* = 0.05) but were higher with the interaction of UFA:SFAxB12 (β = 5.89, 95% CI: 0.36, 11.43, *p* = 0.037) and UFA:SFA × folate (β = 5.61, 95% CI: 0.33, 10.88, *p* = 0.04) in Model 1. See Supplementary Table S4.

**Figure 7:**
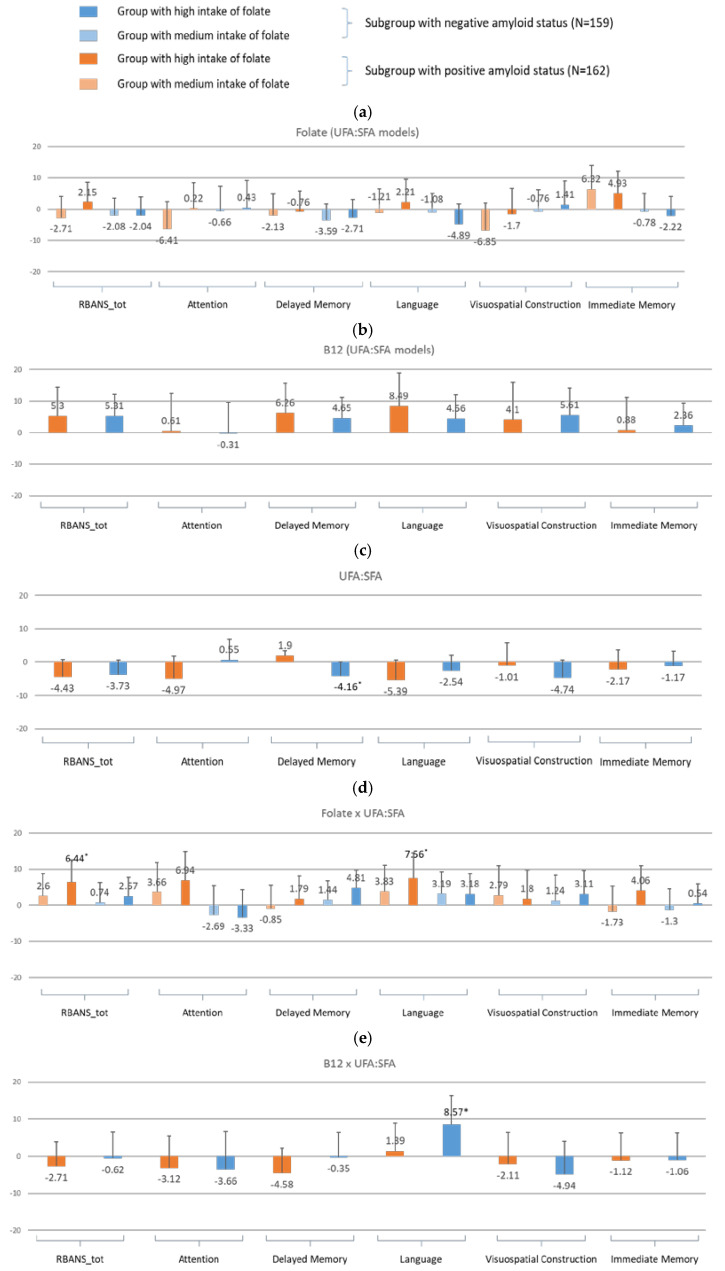
Bar graphs of the estimates for nutritional intake in the amyloid-status-stratified linear regression models with UFA:SFA as one of the predictors for RBANS total and index scores (Model 2) for (**a**) folate, (**b**) vitamin B12, (**c**) UFA:SFA, (**d**) folate × UFA:SFA, (**e**) vitamin B12 × UFA:SFA. Model 2 was adjusted for BMI, education, sex, HDL, LDL, triglycerides, CVD history, and endocrine and metabolic disease history. * indicates statistical significance at *p* < 0.05.

- **For amyloid-specific models** (Figure 7)

When PUFAs were substituted with UFA:SFA in the models, the positive effect of PUFAs on cognitive function among the amyloid-positive group reversed. However, the associations for the amyloid-negative group were weaker than for the PUFA models (delayed memory: β = −4.16, 95% CI: −8.24, −0.07, *p* = 0.05) (Figure 7 vs. Figure 5). The interaction between UFA:SFA × folate reversed most of the negative trends of PUFA × folate in the amyloid-positive group, especially in total RBANS (β = 6.44, 95% CI: 0.34, 12.53, *p* = 0.04) and language (β = 7.56, 95% CI: 0.59, 14.54, *p* = 0.03). In contrast, the positive effects in the amyloid-negative group with PUFA × FA were reduced to non-significant levels. However, the UFA:SFA × B12 models in the amyloid-negative group were associated with higher scores in language (β = 8.57, 95% CI: 0.84, 16.31, *p* = 0.03), as were the three-way interactions of UFA:SFA × FA × B12 (β = −10.65, 95% CI: −19.62, −1.67, *p* < 0.05).

## 4. Discussion

In this cross-sectional study of cognitively healthy older adults, we found varying effects of individual vitamin B12, folate, and PUFA nutrient intakes and their interactions on different cognitive domains depending on participants’ sex and amyloid status; the UFA:SFA ratio, in addition, appears to modulate some of the effects of PUFAs. Our main findings with the amyloid groups were that those in the amyloid-positive group had higher scores across most cognitive domains with a greater PUFA intake, while those in the amyloid-negative group had lower scores in the RBANS total, delayed memory, and visuospatial skills. Vitamin B12 intake was associated with higher scores in both amyloid groups, while folate intake revealed lower RBANS total scores in the amyloid-positive group, especially for males and those with a lower education level, while the amyloid-negative group had significantly higher scores, with similar results for the PUFA × folate interaction. Thus, folate intake may have more benefits for those with a lower AD risk than for those positive for amyloids. For PUFA × B12, there seems to be more of a synergistic effect of these nutrients together for the amyloid-positive group, with trends of higher scores in most domains except for attention; but for the amyloid-negative group, the scores were mostly in the negative direction. So, PUFA × B12 is potentially of benefit to those with a higher AD risk (higher amyloid levels).

Studies have suggested that B vitamins have stronger effects on cognitive function in males than in females—a pattern also observed in our findings. Proposed explanations for this sex-specific stratification include creatinine metabolism, hormonal influences, and genetic factors [43]. While the majority of research highlights the positive association between PUFAs and cognitive function, some studies indicate that elevated levels of PUFAs, particularly omega-6 fatty acids, may impair cognitive performance by promoting neuroinflammation, which corresponds to our observations in the sex-specific models [44], although we did not differentiate the intake of omega-6 from omega-3 in our study. Chang also found that the combination of EPA and DHA intervention provided significant effects on cognitive function in those with mild cognitive impairment, while DHA alone has shown varying results [45,46], which might also explain the findings in our study using all the PUFAs combined.

Our findings support evidence for the interaction between folate and PUFAs in mitigating cognitive decline via the methionine cycle. However, despite the high prevalence of serum B12 deficiency and universally sufficient serum folate levels among our study participants, the J-curve effect on cognition was not clearly evident, with the most benefit sometimes occurring with the highest folate intake and other times with moderate intake. Additionally, there was no negative association between B12 deficiency and cognitive performance in the sex-specific models, contrary to findings in previous studies [47,48]. This discrepancy may result from the use of dietary intake data for folate in the models, which differs from blood-level data due to factors such as malabsorption and food fortification, thereby failing to accurately reflect nutrient status.

When HDL was included in Models 2 and 3, significant associations were found. For example, the positive effect of vitamin B12 on language for all participants was negated in Model 3; whereas the positive association between the interaction of folate, B12, and PUFAs with attention in the amyloid-negative group was only significant in Models 2 and 3, in which the effect of HDL was significant. This suggests that the association between B12 and folate intake and cognition was likely modulated by the concentration of HDL. There are two potential biological pathways. First, HDL helps in the removal of excess cholesterol from cells in a process known as reverse cholesterol transport to ensure adequate cerebral blood flow. Thus, the brain receives sufficient oxygen and nutrients such as B vitamins, and the bioavailability of the vitamins is raised. Moreover, HDL cholesterol is able to reduce oxidative stress and inflammation in blood vessels and to prevent neurodegeneration caused by elevated Hcy levels, which works synergistically with the effects of B vitamins in the methionine cycle, potentially leading to a change in cognitive outcomes [49,50]. While the direct interaction between HDL and B vitamins on cognition is not fully elucidated in the literature, there is strong evidence supporting their individual roles in maintaining cognitive function [50]. Additionally, Hussain et al. linked high HDL levels (>80 mg/dL) to an increased risk of dementia in individuals over 75 [51]. Our study provides new insight, suggesting that moderate HDL cholesterol levels may still offer a protective effect in older adults under the age of 75 with healthy cognitive function.

Several studies have found that the beneficial effects of B-vitamin supplementation were compromised or absent in patients at middle and severe stages of AD with more amyloid accumulation than those with MCI at the mild stage of AD [48]. Our findings are partially comparable to the previous findings. In this study, the negative interference of amyloids with the association between cognition and folate and its interaction with PUFAs were found among the population with relatively lower levels of amyloids. Conversely, both PUFA and B12 intake were more positively associated with cognitive performance in individuals with greater amyloid accumulation in the brain. Since folate and B12 are involved in the methylation cycle through two separate pathways [9,10,11], amyloids could disrupt their bioactivity, respectively, through inhibiting dihydrofolate reductase and oxidizing methionine synthase [52,53]. The gray zone of amyloid levels around the cut-point of SUVR used to allocate the participants in our study as positive or negative may mean that nutrient effects are similar on both sides of the cut-point in the gray zone and the effects on cognition overlap. If we had individual SUVRs for the participants, a sensitivity analysis may have shown where the SUVR cut-point was more sensitive to differences in the effects of nutrient intake on cognition.

In the UFA:SFA models, B vitamins and UFA:SFA were significantly associated with cognitive performance among females, and the associations in the amyloid-status-specific models were partially reversed between the two subgroups. Moreover, this study mostly revealed negative associations between the UFA:SFA ratio and cognition, which conflicts with previous findings [31]. For example, Matura et al. found that a high SFA/(*n*-3) PUFA ratio was significantly correlated with poorer verbal memory performance [54]. This is probably because previous studies did not consider the interaction between nutrients such as B vitamins and the modifying effects between subgroups. However, studies support that there are differences between males and females in how they metabolize and utilize dietary fats under the influence of estrogen and lifestyle factors [55,56] and that SFA influences the formation and aggregation of amyloid plaques among patients with higher levels of amyloids [31,57]. While direct studies comparing UFA:SFA ratios to PUFAs are scarce, existing research highlights the importance of considering the overall quality and composition of dietary fats as a critical determinant of cognitive health [31].

In this study, delayed memory was the cognitive domain most associated with nutrient intake, which aligns with the previous literature. Smith (2016) suggested that low levels of B vitamins were associated with an increased risk of cognitive decline, particularly in memory-related functions [48]. Delayed memory relies heavily on the hippocampus, a brain region that requires efficient methylation, lipid metabolism, and synaptic plasticity for long-term memory storage. The vulnerability of the hippocampus to neuroinflammation, homocysteine toxicity, and phospholipid imbalance means that the disruptions in the nutrients impair delayed memory earlier than other cognitive domains [58]. However, only cognitively healthy individuals were included in this study, and memory and attention tend to be the cognitive domains that are more readily affected during the early stages of cognitive decline or predementia in older adults [59]. In addition, other cognitive domains such as language [60] and attention [61] were also found to be related to deficiency in either B vitamin or PUFA levels by some studies. Conversely, there are multiple studies finding no significant effect of nutrients on any of the cognitive subdomains [62], indicating that more studies are needed to reach a solid conclusion.

Previous studies have shown increased cognitive impairment in RBANS total and delayed memory indices in healthy and preclinical AD for those with a higher amyloid burden [63]. However, to our knowledge, this is the first study, in cognitively healthy adults, to report on the sex- and amyloid-status-stratified associations of the interactions between B vitamins and PUFAs as well as UFA:SFA intake ratios. These differences are important to recognize in clinical settings, as awareness of them can potentially inform nutritional interventions for treatable forms of cognitive impairment. However, the limitations of our study include a relatively high non-response bias, which could lead to a reduced statistical power, and a non-fully represented target population if the excluded participants have different cognitive function levels or dietary patterns compared to those included. The reliance on self-reported dietary data may introduce recall bias, and the cross-sectional nature of the study showing associations does not prove cause and effect. Future research should focus on larger, diverse populations, employ longitudinal designs to validate these findings, and establish causal relationships. Moreover, more precise biomarkers such as Hcy and methyl malonic acid should be measured to establish the true nutrient status, DHA and EPA might be analyzed separately from PUFAs to elucidate their individual effects, and other potentially relevant nutrients such as vitamin D and vitamin B6 should also be included in analytical models to help clarify the associations [45].

## 5. Conclusions

The interaction between PUFAs and B12 may have a beneficial effect on cognitive function in individuals at higher risk of AD, characterized by elevated amyloid β levels, whereas folate intake appears to be more effective in the context of a lower amyloid burden. Sex differences related to other health indicators may also influence responses to B vitamin and PUFA intake. The modifying effects of HDL and LDL cholesterol warrant further investigation, as does the ratio of unsaturated to saturated fatty acid intake.

## Figures and Tables

**Table 1:** Descriptive table by amyloid status.

Variable	Amyloid Positive	Amyloid Negative	*p* Value ^1^
	*n* = 162 (50.57%)	*n* = 159 (49.53%)	
Age (SD ^2^)	72.44 (5.48)	71.09 (5.43)	0.03 *
Sex			
Female	74 (45.70)	80 (50.31)	0.47
Male	88 (54.30)	79 (49.75)	
BMI (SD)	25.99 (4.10)	26.00 (4.05)	0.98
Creatinine (SD)	77.12 (15.19)	75.29 (14.63)	0.27
Triglycerides (IQR ^3^)	1.30 (0.63)	1.36 (0.79)	0.44
Vitamin B12 (blood level) (IQR)	231.60 (96.25)	233.30 (117.50)	0.83
Folate (blood level) (IQR)	31.07 (18.95)	28.29 (14.50)	0.02 *
HDL/mmol/L (IQR)	1.73 (0.58)	1.78 (0.66)	0.37
LDL/mmol/L (IQR)	2.98 (1.35)	3.03 (1.22)	0.63
Education level (%)			0.83
Bachelor	53 (32.72)	50 (31.45)	
Master or higher	47 (29.01)	43 (27.04)	
Secondary school or lower	62 (38.27)	66 (41.51)	
Smoking status (%)			0.13
No	63 (38.89)	70 (44.03)	
Stopped	94 (58.02)	78 (49.05)	
Yes	5 (3.09)	11 (6.92)	
CVD (%)			0.79
Yes	87 (53.70)	82 (51.57)	
No	75 (46.30)	77 (48.43)	
EM (%)			0.45
Yes	34 (20.99)	29 (18.24)	
No	128 (79.01)	130 (81.76)	
APOEε4 carrier (%)			0.00 *
Yes	85 (52.47)	36 (22.64)	
No	77 (47.53)	123 (77.36)	
PUFA/g (IQR)	12.18 (6.35)	12.62 (6.40)	0.50
UFA:SFA ratio (IQR)	1.44 (0.37)	1.45 (0.34)	0.56
Vitamin B12/µg (IQR)	7.14 (3.43)	7.05 (4.60)	0.82
Folate/ug (IQR)	292.20 (159.99)	282.60 (137.26)	0.16
RBANS (SD)	105.50 (12.26)	105.8 (12.26)	0.82
Attention (SD)	109.00 (15.13)	111.10 (16.90)	0.25
Language (SD)	105.00 (14.17)	104.70 (13.00)	0.84
Immediate memory (SD)	108.17 (13.68)	108.50 (12.64)	0.70
Delayed memory (SD)	101.90 (11.91)	102.8 (11.46)	0.45
Visuospatial construction (SD)	96.86 (14.85)	94.74 (15.06)	0.20

^1^ *p* values are from the *t*-tests for normally distributed variables and the Mann–Whitney U test for skewed variables. * *p* < 0.05 indicated differences between groups with positive and negative amyloid status. Abbreviations: ^2^ SD, standard deviation; ^3^ IQR, interquartile range; BMI, body mass index; HDL, high-density lipoprotein; LDL, low-density lipoprotein; CVD, cardiovascular disease; EM, endocrine and metabolic disease; PUFA, polyunsaturated fatty acid; UFA, unsaturated fatty acid, SFA, saturated fatty acid; APOE, apolipoprotein E.

**Table 2:** (**a**) The results of the linear regression models for all participants on RBANS total scores. (**b**) The results of the linear regression models for all participants on the RBANS language subdomain.

(a)						
Nutrients	Model 1 ^1^		Model 2 ^2^		Model 3 ^3^	
	β (95% CI)	*p* Value	β (95% CI)	*p* Value	β (95% CI)	*p* Value
PUFA	−1.69 (−7.80, 4.42)	0.59	−2.32 (−8.52, 3.87)	0.46	−2.21 (−8.47, 4.05)	0.49
B12	3.57 (−3.33, 10.48)	0.31	2.09 (−5.07, 9.25)	0.57	2.06 (−5.20, 9.31)	0.58
Folate, medium	0.15 (−4.46, 4.76)	0.95	0.32 (−4.36, 5.01)	0.89	0.10 (−4.66, 4.87)	0.97
Folate, highest	3.04 (−1.76, 7.84)	0.21	3.66 (−1.24, 8.56)	0.14	3.57 (−1.43, 8.57)	0.16
PUFA: B12	0.77 (−9.04, 10.58)	0.878	−0.55 (−10.48, 9.37)	0.91	−0.84 (−10.87, 9.19)	0.87
PUFA: Folate.medium	2.76 (−4.23, 9.74)	0.44	3.40 (−3.67, 10.47)	0.34	3.18 (−3.97, 10.33)	0.38
PUFA: Folate.high	1.29 (−5.21, 7.79)	0.70	1.72 (−4.84, 8.29)	0.61	1.51 (−5.15, 8.16)	0.66
B12: Folate.medium	−6.24 (−13.91, 1.44)	0.11	−4.76 (−12.75, 3.23)	0.24	−4.89 (−12.96, 3.19)	0.23
B12: Folate.high	−3.54 (−10.66, 3.58)	0.33	−2.25 (−9.59, 5.09)	0.55	−2.19 (−9.62, 5.24)	0.56
PUFA: B12: Folate.medium	−0.77 (−11.17, 9.63)	0.89	0.80 (−9.77, 11.36)	0.88	1.15 (−9.51, 11.82)	0.83
PUFA: B12: Folate.high	−0.36 (−10.31, 9.60)	0.94	1.19 (−8.94, 11.32)	0.82	1.50 (−8.73, 11.74)	0.77
Sex.male	−2.89 (−5.53, −0.25)	0.03 *	−2.41 (−5.89, 1.08)	0.18	−2.18 (−5.78, 1.42)	0.24
BMI	0.24 (−0.09, 0.56)	0.15	0.23 (−0.12, 0.59)	0.20	0.24 (−0.12, 0.61)	0.18
Education.master/higher	0.52 (−2.88, 3.93)	0.76	1.09 (−2.37, 4.54)	0.54	1.24 (−2.26, 4.74)	0.49
Education.secondary	−7.30 (−10.39, −4.21)	0.00 *	−6.72 (−9.88, −3.57)	0.00 *	−6.64 (−9.85, −3.44)	0.00 *
Creatinine			0.01 (−0.10, 0.13)	0.84	0.01 (−0.10, 0.13)	0.85
HDL			1.95 (−1.60, 5.51)	0.28	1.93 (−1.74, 5.59)	0.30
LDL			0.69 (−0.87, 2.24)	0.39	0.77 (−0.80, 2.34)	0.34
Triglycerides			1.44 (−1.05, 3.94)	0.26	1.34 (−1.20, 3.89)	0.30
CVD.yes			1.70 (−1.12, 4.53)	0.24	1.91 (−0.96, 4.78)	0.19
EM.yes			−1.97 (−5.38, 1.43)	0.26	−1.93 (−5.35, 1.49)	0.27
Smokingstatus.yes					0.25 (−6.20, 6.70)	0.94
Smokingstatus.stopped					−1.20 (−4.06, 1.67)	0.41
APOEε4 gene carrier.no					0.55 (−2.26, 3.37)	0.70
**(b)**						
**Nutrients**	**Model 1 ^1^**		**Model 2 ^2^**		**Model 3 ^3^**	
	**β (95% CI)**	***p*** **Value**	**β (95% CI)**	***p*** **Value**	**β (95% CI)**	***p*** **Value**
PUFA	−0.06 (−6.87, 6.76)	0.99	−0.43 (−7.36, 6.51)	0.90	−1.12 (−8.10, 5.87)	0.75
B12	8.86 (1.16, 16.55)	0.02 *	7.92 (−0.11, 15.94)	0.05	7.28 (−0.82, 15.38)	0.08
Folate.medium	−0.52 (−5.66, 4.62)	0.84	−0.02 (−5.27, 5.23)	0.99	0.66 (−4.66, 5.98)	0.81
Folate.high	0.64 (−4.71, 5.99)	0.81	1.29 (−4.19, 6.78)	0.64	1.94 (−3.64, 7.52)	0.49
PUFA × B12	8.98 (−1.96, 19.92)	0.11	8.31 (−2.81, 19.43)	0.14	7.96 (−3.24, 19.15)	0.16
PUFA × Folate.medium	3.32 (−4.47, 11.12)	0.40	3.83 (−4.08, 11.75)	0.34	4.71 (−3.26, 12.69)	0.25
PUFA × Folate.high	−0.89 (−8.14, 6.36)	0.81	−0.65 (−8.00, 6.70)	0.86	0.25 (−7.18, 7.67)	0.95

^1^ Model 1 was adjusted for BMI, education, and sex. ^2^ Model 2 was adjusted for BMI, education, sex, HDL, LDL, triglycerides, CVD history, and endocrine and metabolic disease history. ^3^ Model 3 was adjusted for BMI, education, sex, HDL, LDL, triglycerides, CVD history, endocrine and metabolic disease history, smoking status, and APOE ε4 allele status. * indicates statistical significance at *p* < 0.05. Abbreviations: BMI, body mass index; HDL, high-density lipoprotein; LDL, low-density lipoprotein; CVD, cardiovascular disease; EM, endocrine and metabolic disease; PUFA, polyunsaturated fatty acid; APOE, apolipoprotein E.

## Data Availability

Data and samples collected during the study are not publicly available, due to legal agreements but will be available for collaborative sharing upon application to and approval by the study management group.

## Supplementary Materials

The following supporting information can be downloaded at: https://www.mdpi.com/article/10.3390/nu17091407/s1, Table S1: The results of the linear regression models for all participants on RBANS total and subdomain scores; Table S2: The results of the sex-stratified linear regression models on RBANS total and subdomain scores; Table S3: The results of the amyloid-status-stratified linear regression models on RBANS total and subdomain scores; Table S4: The results of the sex-stratified linear regression models on RBANS total and subdomain scores with UFA:SFA substituting for PUFAs; Table S5: The results of the amyloid-stratified-stratified linear regression models on RBANS total and subdomain scores with UFA:SFA substituting for PUFAs.

## Abbreviations

The following abbreviations are used in this manuscript:

PUFA	Polyunsaturated fatty acid
SCG FFQ	Scottish Collaborative Group—Food Frequency Questionnaire
RBANS	Repeatable Battery for the Assessment of Neuropsychological Status
SFA	Saturated fatty acid
UFA	Unsaturated fatty acid
HDL	High-density lipoprotein
LDL	Low-density lipoprotein
CVD	Cardiovascular disease
EM	Endocrine and metabolic disease
APOE	Apolipoprotein E
PET	Positron emission tomography
CSF	Cerebrospinal fluid
DHA	Docosahexaenoic acid
EPA	Eicosapentaenoic acid
AD	Alzheimer’s disease
Hcy	Homocysteine
MCI	Mild cognitive impairment
*n*-3 FA	Omega-3 fatty acid
PE	Phosphatidylethanolamine
PC	Phosphatidylcholine
PEMT	Phosphatidylethalonamine *N*-methyltransferase
Aβ	Amyloid beta
