# Supplementary material for: Associations Between B Vitamin Interactions with Polyunsaturated Fatty Acids and Cognitive Function Among Cognitively Healthy Older People as Modified by Amyloid Status and Sex
Source: Nutrients. 2025 Apr 23;17(9):1407. doi: 10.3390/nu17091407 (PMC12073187; doi:10.3390/nu17091407)
Supplement: Supplementary file 1 [file nutrients-17-01407-s001.zip › nutrients-3513515-supplementary.pdf]

Table S1. The results of the linear regression models for all participants on RBANS total and subdomain scores.

	Model 1		Model 2		Model 3	
**RBANS domain**	**β (95% CI)**	**P value**	**β (95% CI)**	**P value**	**β (95% CI)**	**P value**
**Attention**						
PUFA	-0.540 (-8.847, 7.767)	0.90	-1.700 (-10.088, 6.689)	0.69	-1.197 (-9.619, 7.226)	0.78
B12	-4.048 (-13.429, 5.333)	0.40	-7.104 (-16.808, 2.599)	0.15	-6.624 (-16.386, 3.137)	0.18
FA.medium	-3.182 (-9.445, 3.081)	0.32	-2.501 (-8.845, 3.844)	0.44	-3.538 (-9.953, 2.876)	0.28
FA.high	-0.459 (-6.978, 6.061)	0.89	0.952 (-5.680, 7.585)	0.78	0.048 (-6.676, 6.773)	0.99
PUFA*B12	-6.323 (-19.652, 7.006)	0.35	-8.772 (-22.218, 4.673)	0.20	-9.211 (-22.704, 4.282)	0.18
PUFA*FA.medium	0.676 (-8.822, 10.173)	0.89	1.948 (-7.622, 11.518)	0.69	1.172 (-8.445, 10.789)	0.81
PUFA*FA.high	-0.138 (-8.971, 8.694)	0.98	0.517 (-8.372, 9.407)	0.91	-0.416 (-9.368, 8.536)	0.93
B12*FA.medium	1.977 (-8.450, 12.404)	0.71	5.227 (-5.593, 16.047)	0.34	4.597 (-6.262, 15.456)	0.41
B12*FA.high	4.155 (-5.523, 13.833)	0.40	6.691 (-3.251, 16.632)	0.19	6.332 (-3.664, 16.328)	0.21
PUFA*B12*FA.medium	6.193 (-7.939, 20.325)	0.39	9.211 (-5.100, 23.522)	0.21	9.764 (-4.583, 24.111)	0.18
PUFA*B12*FA.high	7.491 (-6.036, 21.019)	0.28	10.514 (-3.204, 24.231)	0.13	11.141 (-2.626, 24.908)	0.11
**Delayed memory**						
PUFA	-3.19 (-9.23, 2.85)	0.30	-2.88 (-9.03, 3.28)	0.36	-2.68 (-8.90, 3.54)	0.40
B12	4.23 (-2.59, 11.06)	0.22	3.49 (-3.63, 10.61)	0.34	3.54 (-3.67, 10.75)	0.34
FA.medium	0.29 (-4.26, 4.85)	0.90	0.02 (-4.63, 4.68)	0.99	-0.13 (-4.87, 4.61)	0.96
FA.high	1.76 (-2.98, 6.50)	0.47	1.70 (-3.16, 6.57)	0.49	1.64 (-3.32, 6.61)	0.52
PUFA*B12	-0.19 (-9.88, 9.51)	0.97	-0.68 (-10.54, 9.19)	0.89	-0.72 (-10.69, 9.24)	0.89
PUFA*FA.medium	3.04 (-3.87, 9.95)	0.39	2.75 (-4.27, 9.77)	0.44	2.46 (-4.64, 9.56)	0.50
PUFA*FA.high	4.22 (-2.20, 10.65)	0.20	3.78 (-2.74, 10.30)	0.26	3.53 (-3.08, 10.14)	0.29
B12*FA.medium	-4.34 (-11.93, 3.24)	0.26	-3.45 (-11.38, 4.49)	0.39	-3.67 (-11.69, 4.35)	0.37
B12*FA.high	-5.25 (-12.29, 1.79)	0.14	-4.36 (-11.65, 2.93)	0.24	-4.40 (-11.79, 2.98)	0.24
PUFA*B12*FA.medium	-0.69 (-10.97, 9.58)	0.89	-0.24 (-10.73, 10.26)	0.96	-0.10 (-10.70, 10.49)	0.99
PUFA*B12*FA.high	-0.27 (-10.11, 9.57)	0.96	0.22 (-9.84, 10.28)	0.97	0.25 (-9.91, 10.42)	0.96
**Immediate memory**						
PUFA	0.19 (-6.45, 6.83)	0.96	- 0.25 (-6.99, 6.51)	0.94	0.08 (-6.73, 6.90)	0.98
B12	2.20 (-5.30, 9.70)	0.57	1.76 (-6.05, 9.57)	0.66	2.01 (-5.89, 9.90)	0.62
FA.medium	2.57 (-2.44, 7.57)	0.31	2.81 (-2.30, 7.91)	0.28	2.76 (-2.43, 7.95)	0.3
FA.high	2.60 (-2.62, 7.81)	0.33	3.01 (-2.33, 8.35)	0.27	2.96 (-2.49, 8.40)	0.29
PUFA*B12	2.99 (-7.67, 13.64)	0.58	2.81 (-8.01, 13.62)	0.61	3.27 (-7.65, 14.18)	0.56
PUFA*FA.medium	0.37 (-7.23, 7.96)	0.93	0.87 (-6.83, 8.57)	0.83	0.50 (-7.28, 8.28)	0.9
PUFA*FA.high	-0.81 (-7.87, 6.25)	0.82	-0.33 (-7.48, 6.83)	0.93	-0.61 (-7.86, 6.63)	0.87
B12*FA.medium	-4.28 (-12.61, 4.06)	0.31	-3.94 (-12.65, 4.76)	0.37	-4.33 (-13.11, 4.46)	0.33
B12*FA.high	-1.60 (-9.33, 6.14)	0.68	-1.21 (-9.21, 6.79)	0.77	-1.50 (-9.59, 6.59)	0.72
PUFA*B12*FA.medium	-4.33 (-15.62, 6.97)	0.45	-4.16 (-15.68, 7.35)	0.48	-4.51 (-16.12, 7.10)	0.45
PUFA*B12*FA.high	-2.17 (-12.98, 8.64)	0.69	-1.96 (-12.99, 9.08)	0.73	-2.48 (-13.62, 8.66)	0.66
**Language**						
PUFA	-0.06 (-6.87, 6.76)	0.99	-0.43 (-7.36, 6.51)	0.90	-1.12 (-8.10, 5.87)	0.75
**B12**	**8.86 (1.16, 16.55)**	**0.024 ***	7.92 (-0.11, 15.94)	0.053 .	7.28 (-0.82, 15.38)	0.078 .
FA.medium	-0.52 (-5.66, 4.62)	0.84	-0.02 (-5.27, 5.23)	0.99	0.66 (-4.66, 5.98)	0.81
FA.high	0.64 (-4.71, 5.99)	0.81	1.29 (-4.19, 6.78)	0.64	1.94 (-3.64, 7.52)	0.49
PUFA*B12	8.98 (-1.96, 19.92)	0.11	8.31 (-2.81, 19.43)	0.14	7.96 (-3.24, 19.15)	0.16
PUFA*FA.medium	3.32 (-4.47, 11.12)	0.40	3.83 (-4.08, 11.75)	0.34	4.71 (-3.26, 12.69)	0.25
PUFA*FA.high	-0.89 (-8.14, 6.36)	0.81	-0.65 (-8.00, 6.70)	0.86	0.25 (-7.18, 7.67)	0.95
**B12*FA.medium**	**-12.31 (-20.86, -3.75)**	**0.005 ****	**-11.50 (-20.45, -2.55)**	**0.012 ***	**-10.66 (-19.67, -1.65)**	**0.021 ***
**B12*FA.high**	**-8.51 (-16.45, -0.57)**	**0.036 ***	-7.93 (-16.16, 0.29)	0.059 .	-7.32 (-15.62, 0.97)	0.083 .

PUFA*B12*FA.medium	-5.93 (-17.53, 5.66)	0.32	-4.92 (-16.75, 6.91)	0.41	-4.75 (-16.65, 7.15)	0.43
PUFA*B12*FA.high	-8.88 (-19.98, 2.22)	0.12	-7.98 (-19.33, 3.36)	0.167	-7.67 (-19.09, 3.75)	0.19
**Visuospatial construction**						
PUFA	-3.50 (-11.31, 4.30)	0.38	-4.06 (-11.96, 3.83)	0.31	-4.10 (-12.09, 3.88)	0.31
B12	1.49 (-7.32, 10.31)	0.74	1.48 (-7.65, 10.61)	0.75	1.33 (-7.93, 10.59)	0.78
FA.medium	2.15 (-3.74, 8.03)	0.47	1.38 (-4.59, 7.35)	0.65	1.41 (-4.67, 7.50)	0.65
FA.high	4.66 (-1.46, 10.79)	0.14	4.26 (-1.98, 10.49)	0.18	4.37 (-2.01, 10.75)	0.18
PUFA*B12	-2.45 (-14.98, 10.07)	0.70	-3.35 (-16.00, 9.29)	0.60	-3.55 (-16.35, 9.24)	0.59
PUFA*FA.medium	3.41 (-5.51, 12.33)	0.45	3.57 (-5.44, 12.57)	0.44	3.58 (-5.54, 12.70)	0.44
PUFA*FA.high	3.61 (-4.69, 11.91)	0.39	4.18 (-4.18, 12.54)	0.33	4.21 (-4.27, 12.70)	0.33
B12*FA.medium	-2.21 (-12.01, 7.59)	0.66	-2.23 (-12.40, 7.95)	0.67	-2.16 (-12.46, 8.13)	0.68
B12*FA.high	-0.60 (-9.69, 8.50)	0.90	-0.30 (-9.66, 9.05)	0.95	-0.15 (-9.63, 9.33)	0.98
PUFA*B12*FA.medium	1.32 (-11.96, 14.60)	0.85	2.06 (-11.40, 15.52)	0.76	2.28 (-11.33, 15.88)	0.74
PUFA*B12*FA.high	2.51 (-10.20, 15.22)	0.70	3.31 (-9.60, 16.21)	0.61	3.50 (-9.56, 16.55)	0.60
**Total RBANS**						
PUFA	-1.69 (-7.80, 4.42)	0.59	-2.32 (-8.52, 3.87)	0.46	-2.21 (-8.47, 4.05)	0.49
B12	3.57 (-3.33, 10.48)	0.31	2.09 (-5.07, 9.25)	0.57	2.06 (-5.20, 9.31)	0.58
FA.medium	0.15 (-4.46, 4.76)	0.95	0.32 (-4.36, 5.01)	0.89	0.10 (-4.66, 4.87)	0.97
FA.high	3.04 (-1.76, 7.84)	0.21	3.66 (-1.24, 8.56)	0.14	3.57 (-1.43, 8.57)	0.16
PUFA*B12	0.77 (-9.04, 10.58)	0.88	-0.55 (-10.48, 9.37)	0.91	-0.84 (-10.87, 9.19)	0.87
PUFA*FA.medium	2.76 (-4.23, 9.74)	0.44	3.40 (-3.67, 10.47)	0.34	3.18 (-3.97, 10.33)	0.38
PUFA*FA.high	1.29 (-5.21, 7.79)	0.70	1.72 (-4.84, 8.29)	0.61	1.51 (-5.15, 8.16)	0.66
B12*FA.medium	-6.24 (-13.91, 1.44)	0.11	-4.76 (-12.75, 3.23)	0.24	-4.89 (-12.96, 3.19)	0.23
B12*FA.high	-3.54 (-10.66, 3.58)	0.33	-2.25 (-9.59, 5.09)	0.55	-2.19 (-9.62, 5.24)	0.56
PUFA*B12*FA.medium	-0.77 (-11.17, 9.63)	0.89	0.80 (-9.77, 11.36)	0.88	1.15 (-9.51, 11.82)	0.83
PUFA*B12*FA.high	-0.36 (-10.31, 9.60)	0.94	1.19 (-8.94, 11.32)	0.82	1.50 (-8.73, 11.74)	0.77

Results with p<0.05 are shown in bold with *

Table S2. The results of the sex-stratified linear regression models on RBANS total and subdomain scores.

	Female			Male		
	**Model 1**	**Model 2**	**Model 3**	**Model 1**	**Model 2**	**Model 3**
**RBANS domain**	**β (95% CI)**					
**Attention**						
PUFA	2.91 (-6.93, 12.74)	2.26 (-7.80, 12.32)	3.06 (-7.21, 13.34)	-12.27 (-30.09, 5.55)	-13.34 (-31.98, 5.29)	-14.04 (-32.49, 4.40)
B12	-5.50 (-17.20, 6.20)	-10.12 (-22.98, 2.73)	-8.23 (-21.27, 4.81)	-7.28 (-25.43, 10.86)	-9.30 (-28.03, 9.43)	-11.08 (-29.74, 7.58)
FA.medium	-4.09 (-12.45, 4.28)	-3.64 (-12.30, 5.02)	-4.81 (-13.73, 4.10)	1.05 (-10.49, 12.59)	1.58 (-10.53, 13.70)	1.62 (-10.37, 13.61)
FA.high	-4.51 (-13.38, 4.35)	-2.33 (-11.54, 6.88)	-3.88 (-13.31, 5.55)	5.48 (-6.42, 17.38)	6.12 (-6.15, 18.39)	6.20 (-5.99, 18.39)
PUFA*B12	-3.16 (-19.63, 13.32)	-6.33 (-23.29, 10.63)	-4.49 (-21.89, 12.90)	-21.09 (-48.28, 6.10)	-22.61 (-50.82, 5.61)	-24.66 (-52.62, 3.31)
PUFA*FA.medium	-3.51 (-15.30, 8.28)	-2.54 (-14.48, 9.41)	-3.45 (-15.55, 8.65)	16.20 (-3.33, 35.74)	17.09 (-3.27, 37.46)	18.49 (-1.78, 38.77)
PUFA*FA.high	-2.27 (-12.94, 8.40)	-2.51 (-13.41, 8.39)	-3.69 (-14.86, 7.48)	11.86 (-6.62, 30.34)	12.71 (-6.61, 32.02)	12.99 (-6.14, 32.12)
B12*FA.medium	4.56 (-9.28, 18.40)	9.84 (-5.53, 25.22)	7.49 (-8.18, 23.15)	5.18 (-13.94, 24.31)	7.43 (-12.26, 27.12)	8.83 (-10.79, 28.45)
B12*FA.high	5.52 (-8.01, 19.04)	9.72 (-4.81, 24.26)	7.98 (-6.70, 22.65)	6.84 (-11.50, 25.17)	8.61 (-10.13, 27.35)	10.46 (-8.20, 29.13)
PUFA*B12*FA.medium	5.29 (-12.90, 23.49)	9.67 (-9.18, 28.51)	7.83 (-11.48, 27.15)	17.13 (-10.98, 45.25)	18.84 (-10.49, 48.18)	20.07 (-8.95, 49.10)
PUFA*B12*FA.high	9.21 (-8.20, 26.61)	13.05 (-5.04, 31.15)	11.83 (-6.64, 30.31)	20.92 (-6.41, 48.26)	22.74 (-5.68, 51.16)	25.02 (-3.15, 53.20)
**Delayed memory**						

PUFA	-3.42 (-10.14, 3.29)	-3.75 (-10.60, 3.09)	-3.99 (-11.05, 3.06)	-6.19 (-19.80, 7.43)	-6.52 (-20.78, 7.73)	-6.26 (-20.59, 8.06)
B12	6.52 (-1.47, 14.51)	6.03 (-2.71, 14.77)	6.14 (-2.82, 15.10)	-1.63 (-15.50, 12.24)	-3.23 (-17.55, 11.10)	-2.49 (-16.98, 12.00)
FA.medium	0.28 (-5.44, 5.99)	-0.19 (-6.08, 5.70)	-0.27 (-6.39, 5.86)	0.57 (-8.24, 9.39)	0.72 (-8.55, 9.99)	0.45 (-8.87, 9.76)
FA.high	0.61 (-5.44, 6.67)	-0.11 (-6.38, 6.15)	-0.12 (-6.60, 6.36)	4.41 (-4.68, 13.50)	4.54 (-4.85, 13.92)	4.21 (-5.26, 13.68)
PUFA*B12	2.96 (-8.29, 14.21)	1.96 (-9.58, 13.49)	1.61 (-10.35, 13.56)	-9.72 (-30.50, 11.06)	-12.21 (-33.80, 9.37)	-11.95 (-33.67, 9.77)
PUFA*FA.medium	2.44 (-5.61, 10.49)	2.41 (-5.72, 10.53)	2.50 (-5.82, 10.81)	5.31 (-9.62, 20.24)	5.50 (-10.08, 21.08)	4.65 (-11.10, 20.40)
PUFA*FA.high	5.76 (-1.53, 13.04)	6.30 (-1.11, 13.71)	6.48 (-1.20, 14.15)	6.21 (-7.91, 20.33)	6.68 (-8.10, 21.45)	6.17 (-8.69, 21.03)
B12*FA.medium	-11.48 (-20.94, -2.03)	-10.68 (-21.13, -0.22)	-10.71 (-21.47, 0.06)	4.60 (-10.02, 19.22)	5.82 (-9.24, 20.88)	5.12 (-10.12, 20.35)
B12*FA.high	-9.86 (-19.09, -0.62)	-8.88 (-18.77, 1.00)	-8.98 (-19.06, 1.10)	0.69 (-13.32, 14.70)	2.18 (-12.16, 16.51)	1.51 (-12.99, 16.00)
PUFA*B12*FA.medium	-6.52 (-18.94, 5.91)	-6.26 (-19.07, 6.56)	-5.88 (-19.15, 7.39)	9.88 (-11.61, 31.36)	12.74 (-9.70, 35.19)	12.87 (-9.68, 35.41)
PUFA*B12*FA.high	-1.11 (-13.00, 10.77)	0.18 (-12.13, 12.48)	0.67 (-12.02, 13.36)	9.20 (-11.69, 30.08)	11.57 (-10.17, 33.31)	11.40 (-10.49, 33.28)
**Immediate memory**						
PUFA	1.60 (-5.85, 9.06)	-0.43 (-7.73, 6.88)	0.05 (-7.46, 7.56)	-7.61 (-22.43, 7.21)	-9.64 (-25.11, 5.83)	-9.28 (-24.80, 6.23)
B12	3.45 (-5.41, 12.32)	4.09 (-5.24, 13.42)	4.90 (-4.64, 14.43)	-4.63 (-19.72, 10.46)	-7.66 (-23.20, 7.88)	-6.67 (-22.36, 9.03)
FA.medium	-0.68 (-7.02, 5.66)	-0.79 (-7.08, 5.49)	-1.18 (-7.70, 5.33)	7.82 (-1.78, 17.41)	9.08 (-0.98, 19.13)	8.74 (-1.35, 18.82)
FA.high	0.17 (-6.55, 6.88)	0.50 (-6.19, 7.19)	-0.18 (-7.07, 6.71)	8.08 (-1.81, 17.98)	9.24 (-0.94, 19.43)	8.85 (-1.41, 19.10)
PUFA*B12	5.11 (-7.38, 17.60)	4.39 (-7.92, 16.70)	5.62 (-7.10, 18.34)	-8.94 (-31.55, 13.68)	-13.28 (-36.70, 10.14)	-12.87 (-36.39, 10.65)
PUFA*FA.medium	-2.13 (-11.06, 6.81)	-0.95 (-9.62, 7.73)	-1.34 (-10.19, 7.51)	8.77 (-7.48, 25.01)	10.95 (-5.96, 27.85)	9.82 (-7.23, 26.88)
PUFA*FA.high	-1.63 (-9.72, 6.45)	0.78 (-7.13, 8.70)	0.21 (-7.96, 8.37)	7.32 (-8.05, 22.69)	9.49 (-6.54, 25.52)	8.84 (-7.25, 24.93)
B12*FA.medium	**-12.02 (-22.50, -1.53)***	**-13.60 (-24.76, -2.44)***	**-14.62 (-26.08, -3.17)***	6.71 (-9.19, 22.62)	9.24 (-7.10, 25.58)	8.28 (-8.22, 24.79)
B12*FA.high	-2.97 (-13.22, 7.28)	-2.72 (-13.27, 7.83)	-3.47 (-14.20, 7.25)	4.81 (-10.43, 20.06)	7.31 (-8.25, 22.86)	6.39 (-9.31, 22.09)
PUFA*B12*FA.medium	-8.69 (-22.48, 5.10)	-9.04 (-22.72, 4.64)	-10.32 (-24.44, 3.79)	6.49 (-16.89, 29.87)	11.57 (-12.78, 35.92)	11.68 (-12.73, 36.09)
PUFA*B12*FA.high	-1.72 (-14.91, 11.48)	-1.22 (-14.36, 11.91)	-2.22 (-15.72, 11.29)	8.97 (-13.77, 31.70)	13.36 (-10.23, 36.95)	13.04 (-10.65, 36.74)
**Language**						
PUFA	2.18 (-5.66, 10.02)	2.27 (-5.67, 10.20)	1.56 (-6.59, 9.72)	-5.65 (-20.63, 9.32)	-8.75 (-24.36, 6.86)	-8.82 (-24.51, 6.87)
B12	8.93 (-0.40, 18.26)	6.93 (-3.21, 17.06)	7.04 (-3.31, 17.40)	7.44 (-7.80, 22.69)	4.59 (-11.11, 20.28)	4.35 (-11.51, 20.22)
FA.medium	0.22 (-6.45, 6.89)	2.12 (-4.71, 8.95)	2.31 (-4.76, 9.39)	0.56 (-9.14, 10.25)	2.52 (-7.63, 12.67)	2.77 (-7.43, 12.97)
FA.high	1.01 (-6.06, 8.08)	2.80 (-4.46, 10.06)	2.98 (-4.51, 10.46)	0.37 (-9.63, 10.37)	1.47 (-8.81, 11.75)	1.83 (-8.54, 12.20)
PUFA*B12	7.81 (-5.33, 20.95)	8.04 (-5.33, 21.42)	7.64 (-6.17, 21.44)	7.19 (-15.66, 30.03)	14.82 (-2.24, 31.89)	2.20 (-21.59, 25.98)
PUFA*FA.medium	-1.00 (-10.40, 8.39)	-0.85 (-10.27, 8.57)	-0.37 (-9.97, 9.24)	12.38 (-4.04, 28.79)	10.34 (-5.84, 26.53)	15.19 (-2.05, 32.43)
PUFA*FA.high	-4.77 (-13.28, 3.74)	-5.06 (-13.66, 3.53)	-4.38 (-13.25, 4.49)	7.33 (-8.20, 22.85)	-7.93 (-24.42, 8.57)	10.70 (-5.57, 26.96)
B12*FA.medium	-12.66 (-23.69, -1.62)	-10.78 (-22.90, 1.34)	-10.56 (-22.99, 1.88)	-11.06 (-27.13, 5.01)	-5.14 (-20.84, 10.56)	-7.74 (-24.42, 8.95)
B12*FA.high	-6.68 (-17.46, 4.10)	-4.62 (-16.08, 6.84)	-4.74 (-16.39, 6.90)	-7.43 (-22.84, 7.97)	-0.83 (-25.41, 23.75)	-4.98 (-20.85, 10.90)
PUFA*B12*FA.medium	-4.70 (-19.21, 9.80)	-4.41 (-19.28, 10.45)	-4.04 (-19.37, 11.29)	-6.58 (-30.20, 17.04)	-2.66 (-26.47, 21.15)	-1.16 (-25.85, 23.52)
PUFA*B12*FA.high	-8.09 (-21.97, 5.79)	-8.81 (-23.08, 5.46)	-8.04 (-22.70, 6.62)	-8.14 (-31.11, 14.83)	0.04 (-0.59, 0.68)	-3.03 (-26.99, 20.93)
**Visuospatial construction**						
PUFA	0.58 (-8.46, 9.63)	-0.75 (-9.95, 8.46)	-2.31 (-11.70, 7.07)	-12.37 (-29.15, 4.41)	-12.70 (-30.00, 4.61)	-12.27 (-29.63, 5.10)
B12	-0.99 (-11.76, 9.76)	-4.28 (-16.04, 7.48)	-5.09 (-17.01, 6.82)	3.04 (-14.05, 20.13)	2.41 (-14.99, 19.80)	3.59 (-13.97, 21.15)
FA.medium	3.62 (-4.07, 11.32)	3.10 (-4.82, 11.03)	3.57 (-4.57, 11.72)	4.68 (-6.19, 15.54)	4.21 (-7.04, 15.46)	3.79 (-7.50, 15.08)
FA.high	3.67 (-4.48, 11.83)	4.72 (-3.70, 13.15)	5.74 (-2.87, 14.36)	8.94 (-2.27, 20.15)	8.09 (-3.31, 19.48)	7.48 (-3.99, 18.95)
PUFA*B12	-5.99 (-21.15, 9.16)	-9.46 (-24.98, 6.05)	-12.21 (-28.10, 3.69)	-1.48 (-27.08, 24.13)	-3.97 (-30.17, 22.24)	-3.45 (-29.77, 22.88)
PUFA*FA.medium	-1.37 (-12.21, 9.47)	-0.31 (-11.24, 10.62)	0.69 (-10.37, 11.74)	15.47 (-2.93, 33.86)	14.51 (-4.41, 33.42)	13.28 (-5.81, 32.37)
PUFA*FA.high	-1.91 (-11.73, 7.90)	-1.01 (-10.99, 8.96)	0.53 (-9.67, 10.74)	12.88 (-4.53, 30.28)	13.86 (-4.08, 31.80)	13.17 (-4.84, 31.18)
B12*FA.medium	2.45 (-10.28, 15.18)	5.94 (-8.12, 20.00)	7.41 (-6.90, 21.73)	-5.39 (-23.40, 12.62)	-4.88 (-23.17, 13.40)	-5.83 (-24.30, 12.64)

B12*FA.high	3.29 (-9.15, 15.72)	6.78 (-6.52, 20.08)	7.56 (-5.85, 20.97)	-2.19 (-19.46, 15.07)	-1.14 (-18.54, 16.27)	-2.25 (-19.81, 15.32)
PUFA*B12*FA.medium	6.84 (-9.89, 23.58)	10.37 (-6.87, 27.61)	13.23 (-4.42, 30.87)	-2.16 (-28.64, 24.32)	0.06 (-27.19, 27.31)	0.04 (-27.28, 27.36)
PUFA*B12*FA.high	2.35 (-13.66, 18.35)	6.58 (-9.97, 23.14)	9.48 (-7.40, 26.36)	2.00 (-23.74, 27.75)	3.83 (-22.56, 30.23)	3.39 (-23.13, 29.90)
**Total RBANS**						
PUFA	1.33 (-5.97, 8.64)	0.13 (-7.28, 7.53)	-0.21 (-7.81, 7.38)	3.74 (-4.65, 12.13)	**-13.97 (-27.39, -0.55)***	**-13.93 (-27.39, -0.48)***
B12	3.41 (-5.27, 12.10)	0.66 (-8.80, 10.12)	1.25 (-8.39, 10.89)	-0.68 (-13.88, 12.51)	-3.57 (-17.05, 9.91)	-3.43 (-17.04, 10.18)
FA.medium	-0.31 (-6.53, 5.90)	0.09 (-6.28, 6.47)	-0.34 (-6.93, 6.25)	3.74 (-4.65, 12.13)	4.76 (-3.96, 13.48)	4.55 (-4.19, 13.30)
FA.high	0.34 (-6.25, 6.92)	1.77 (-5.01, 8.55)	1.44 (-5.53, 8.42)	8.45 (-0.21, 17.10)	**9.08 (0.25, 17.91)***	8.86 (-0.04, 17.75)
PUFA*B12	1.52 (-10.72, 13.75)	-0.74 (-13.22, 11.74)	-1.23 (-14.09, 11.64)	-9.19 (-28.97, 10.58)	-13.85 (-34.16, 6.47)	-14.13 (-34.54, 6.27)
PUFA*FA.medium	-2.24 (-10.99, 6.52)	-1.27 (-10.06, 7.52)	-1.29 (-10.24, 7.66)	**16.63 (2.43, 30.84)***	**18.02 (3.35, 32.68)***	**17.69 (2.90, 32.48)***
PUFA*FA.high	-1.52 (-9.44, 6.41)	-0.60 (-8.62, 7.43)	-0.48 (-8.74, 7.78)	12.17 (-1.27, 25.61)	**14.34 (0.44, 28.25)***	**14.02 (0.06, 27.97)***
B12*FA.medium	-8.63 (-18.91, 1.65)	-5.89 (-17.20, 5.43)	-6.42 (-18.00, 5.16)	-0.43 (-14.34, 13.47)	2.26 (-11.91, 16.43)	2.05 (-12.27, 16.36)
B12*FA.high	-3.03 (-13.07, 7.02)	0.04 (-10.66, 10.74)	-0.49 (-11.34, 10.36)	0.28 (-13.06, 13.61)	2.86 (-10.63, 16.35)	2.79 (-10.83, 16.40)
PUFA*B12*FA.medium	-1.75 (-15.26, 11.76)	0.55 (-13.32, 14.42)	1.11 (-13.17, 15.39)	6.32 (-14.13, 26.76)	11.41 (-9.71, 32.53)	11.81 (-9.37, 32.98)
PUFA*B12*FA.high	0.72 (-12.20, 13.65)	3.24 (-10.08, 16.56)	4.11 (-9.55, 17.77)	8.81 (-11.07, 28.69)	13.39 (-7.06, 33.85)	13.81 (-6.74, 34.36)

Results with p<0.05 are indicated in bold with *

Table S3. The results of the amyloid-status-stratified linear regression models on RBANS total and subdomain scores.

	**Positive**			**Negative**		
	**1**	**2**	**3**	**1**	**2**	**3**
**RBANS domain**	**β (95% CI)**					
**Attention**						
PUFA	-6.68 (-18.68, 5.32)	3.61 (-11.08, 18.30)	4.12 (-10.31, 18.56)	-6.68 (-18.68, 5.32)	-7.88 (-19.86, 4.10)	-8.47 (-20.65, 3.71)
B12	-1.79 (-13.42, 9.83)	-9.06 (-30.10, 11.98)	-7.73 (-28.37, 12.91)	-1.79 (-13.42, 9.83)	-6.51 (-18.75, 5.73)	-6.52 (-18.93, 5.90)
FA.medium	0.23 (-8.65, 9.11)	-6.24 (-17.42, 4.93)	-7.80 (-18.75, 3.14)	0.23 (-8.65, 9.11)	2.08 (-6.88, 11.05)	1.96 (-7.18, 11.09)
FA.high	0.20 (-9.92, 10.32)	-0.18 (-11.04, 10.67)	-3.07 (-13.80, 7.66)	0.20 (-9.92, 10.32)	3.01 (-7.23, 13.25)	3.34 (-7.00, 13.68)
PUFA*B12	-12.69 (-30.70, 5.31)	-3.44 (-30.31, 23.42)	-5.79 (-32.28, 20.71)	-12.69 (-30.70, 5.31)	-16.36 (-34.41, 1.68)	-16.89 (-35.29, 1.51)
PUFA*FA.medium	9.35 (-4.52, 23.22)	-5.84 (-22.01, 10.34)	-7.76 (-23.72, 8.20)	9.35 (-4.52, 23.22)	11.86 (-2.01, 25.73)	12.33 (-1.71, 26.37)
PUFA*FA.high	5.22 (-7.61, 18.05)	-3.22 (-18.74, 12.30)	-4.74 (-20.01, 10.53)	5.22 (-7.61, 18.05)	5.63 (-7.12, 18.37)	6.05 (-6.96, 19.06)
B12*FA.medium	-1.92 (-15.23, 11.39)	9.89 (-12.35, 32.13)	9.20 (-12.65, 31.06)	-1.92 (-15.23, 11.39)	2.79 (-11.27, 16.84)	2.75 (-11.43, 16.94)
B12*FA.high	0.16 (-14.65, 14.96)	7.99 (-13.17, 29.16)	6.95 (-13.81, 27.71)	0.16 (-14.65, 14.96)	4.68 (-10.61, 19.96)	4.77 (-10.75, 20.29)
PUFA*B12*FA.medium	11.63 (-7.54, 30.80)	4.57 (-23.19, 32.32)	5.89 (-21.36, 33.15)	11.63 (-7.54, 30.80)	15.19 (-4.08, 34.45)	15.66 (-3.93, 35.24)
PUFA*B12*FA.high	15.21 (-3.63, 34.06)	4.34 (-22.71, 31.39)	7.05 (-19.65, 33.76)	15.21 (-3.63, 34.06)	19.05 (0.14, 37.97)	19.80 (0.63, 38.96)
**Delayed memory**						
PUFA	9.26 (-1.87, 20.38)	**17.96 (1.31, 34.61)***	7.43 (-4.29, 19.15)	**-9.71 (-17.60, -1.83)***	**-10.03 (-17.90, -2.15)***	**-9.68 (-17.64, -1.72)***
B12	**17.61 (1.37, 33.86)***	8.07 (-3.55, 19.69)	16.42 (-0.33, 33.18)	2.97 (-4.66, 10.61)	0.71 (-7.33, 8.75)	0.71 (-7.40, 8.82)
FA.medium	-4.84 (-13.26, 3.58)	-4.51 (-13.35, 4.33)	-4.19 (-13.07, 4.70)	0.67 (-5.16, 6.50)	1.55 (-4.34, 7.44)	1.81 (-4.16, 7.78)
FA.high	-3.23 (-11.55, 5.08)	-3.11 (-11.69, 5.48)	-2.54 (-11.25, 6.17)	0.89 (-5.75, 7.54)	0.98 (-5.75, 7.71)	0.78 (-5.98, 7.53)
PUFA*B12	**21.57 (0.84, 42.31)***	20.38 (-0.87, 41.64)	18.18 (-3.34, 39.69)	-8.50 (-20.32, 3.32)	-10.00 (-21.86, 1.86)	-9.60 (-21.62, 2.42)
PUFA*FA.medium	-7.18 (-19.34, 4.97)	-5.68 (-18.48, 7.11)	-5.32 (-18.28, 7.64)	7.24 (-1.87, 16.35)	8.54 (-0.58, 17.65)	8.29 (-0.89, 17.46)
PUFA*FA.high	-7.88 (-19.57, 3.81)	-6.49 (-18.77, 5.79)	-6.02 (-18.42, 6.37)	**11.07 (2.65, 19.49)***	**11.41 (3.04, 19.78)***	**11.23 (2.73, 19.73)***
B12*FA.medium	**-20.93 (-38.11, -3.75)***	**-21.49 (-39.09, -3.89)***	**-19.65 (-37.40, -1.90)***	-0.37 (-9.11, 8.37)	1.98 (-7.26, 11.21)	2.16 (-7.11, 11.44)
B12*FA.high	**-19.72 (-36.08, -3.35)***	**-20.16 (-36.90, -3.41)***	**-18.54 (-35.40, -1.68)***	0.43 (-9.29, 10.15)	3.32 (-6.73, 13.36)	3.24 (-6.90, 13.39)
PUFA*B12*FA.medium	**-26.49 (-47.93, -5.06)***	**-25.45 (-47.41, -3.49)***	**-23.49 (-45.63, -1.36)***	11.14 (-1.45, 23.73)	12.11 (-0.55, 24.77)	11.65 (-1.15, 24.45)

PUFA*B12*FA.high	**-23.34 (-44.22, -2.46)***	**-22.32 (-43.72, -0.91)***	-19.99 (-41.67, 1.70)	8.30 (-4.07, 20.68)	9.70 (-2.73, 22.13)	9.14 (-3.38, 21.67)
**Immediate memory**						
PUFA	4.86 (-7.76, 17.48)	3.60 (-9.53, 16.73)	4.34 (-8.99, 17.66)	-2.85 (-11.45, 5.75)	-2.87 (-11.50, 5.77)	-3.46 (-12.21, 5.29)
B12	6.45 (-11.98, 24.88)	6.57 (-12.24, 25.37)	7.60 (-11.45, 26.65)	1.69 (-6.64, 10.02)	1.48 (-7.34, 10.30)	0.95 (-7.97, 9.87)
FA.medium	3.52 (-6.03, 13.07)	4.31 (-5.68, 14.30)	3.63 (-6.47, 13.74)	1.37 (-4.99, 7.74)	1.25 (-5.21, 7.70)	2.07 (-4.49, 8.63)
FA.high	2.58 (-6.85, 12.01)	3.20 (-6.50, 12.90)	2.20 (-7.70, 12.10)	0.44 (-6.81, 7.69)	0.45 (-6.93, 7.82)	0.76 (-6.67, 8.19)
PUFA*B12	11.53 (-12.00, 35.05)	11.01 (-13.01, 35.03)	11.40 (-13.06, 35.85)	-1.14 (-14.05, 11.76)	-1.95 (-14.95, 11.05)	-2.81 (-16.03, 10.41)
PUFA*FA.medium	-3.60 (-17.39, 10.19)	-1.83 (-16.29, 12.63)	-3.05 (-17.78, 11.69)	2.06 (-7.88, 12.00)	2.54 (-7.46, 12.53)	3.14 (-6.94, 13.23)
PUFA*FA.high	-2.58 (-15.84, 10.68)	-1.51 (-15.39, 12.37)	-2.54 (-16.63, 11.56)	1.24 (-7.95, 10.43)	1.45 (-7.73, 10.62)	2.38 (-6.97, 11.72)
B12*FA.medium	-8.74 (-28.23, 10.75)	-8.81 (-28.69, 11.07)	-9.80 (-29.97, 10.37)	-3.16 (-12.70, 6.38)	-3.82 (-13.95, 6.30)	-3.11 (-13.30, 7.08)
B12*FA.high	-7.20 (-25.76, 11.37)	-7.81 (-26.73, 11.11)	-8.75 (-27.91, 10.41)	3.55 (-7.06, 14.16)	3.66 (-7.35, 14.67)	4.33 (-6.82, 15.48)
PUFA*B12*FA.medium	-17.52 (-41.83, 6.79)	-16.67 (-41.48, 8.14)	-17.23 (-42.38, 7.93)	3.18 (-10.55, 16.92)	3.46 (-10.42, 17.33)	4.00 (-10.07, 18.07)
PUFA*B12*FA.high	-12.84 (-36.53, 10.84)	-12.05 (-36.24, 12.13)	-12.35 (-37.00, 12.30)	2.34 (-11.16, 15.85)	2.70 (-10.93, 16.32)	3.30 (-10.47, 17.07)
**Language**						
PUFA	5.40 (-7.53, 18.34)	3.90 (-9.52, 17.33)	2.91 (-10.67, 16.50)	-4.10 (-13.08, 4.88)	-4.90 (-14.02, 4.23)	5.40 (-7.53, 18.34)
B12	14.25 (-4.64, 33.14)	12.79 (-6.43, 32.02)	11.49 (-7.93, 30.91)	6.99 (-2.19, 16.16)	6.59 (-2.72, 15.89)	14.25 (-4.64, 33.14)
FA.medium	-2.05 (-11.84, 7.74)	-1.72 (-11.93, 8.49)	-0.87 (-11.17, 9.43)	1.69 (-5.03, 8.41)	2.43 (-4.42, 9.27)	-2.05 (-11.84, 7.74)
FA.high	1.08 (-8.59, 10.74)	1.50 (-8.42, 11.42)	2.72 (-7.37, 12.82)	-1.47 (-9.14, 6.21)	-1.05 (-8.80, 6.70)	1.08 (-8.59, 10.74)
PUFA*B12	15.33 (-8.79, 39.44)	12.81 (-11.74, 37.36)	12.24 (-12.69, 37.17)	6.33 (-7.19, 19.86)	5.46 (-8.33, 19.25)	15.33 (-8.79, 39.44)
PUFA*FA.medium	-5.26 (-19.39, 8.87)	-3.94 (-18.72, 10.84)	-2.34 (-17.36, 12.68)	10.32 (-0.08, 20.71)	**11.08 (0.56, 21.60)***	-5.26 (-19.39, 8.87)
PUFA*FA.high	-5.23 (-18.82, 8.36)	-3.48 (-17.67, 10.70)	-2.14 (-16.51, 12.23)	3.26 (-6.29, 12.81)	4.29 (-5.46, 14.04)	-5.23 (-18.82, 8.36)
B12*FA.medium	-13.88 (-33.86, 6.09)	-12.88 (-33.21, 7.45)	-11.62 (-32.18, 8.95)	**-13.30 (-23.83, -2.76)***	**-12.60 (-23.24, -1.97)***	-13.88 (-33.86, 6.09)
B12*FA.high	-14.41 (-33.44, 4.62)	-13.15 (-32.50, 6.19)	-11.97 (-31.50, 7.57)	-6.60 (-18.06, 4.85)	-6.07 (-17.70, 5.56)	-14.41 (-33.44, 4.62)
PUFA*B12*FA.medium	-12.86 (-37.78, 12.06)	-10.33 (-35.69, 15.04)	-9.56 (-35.21, 16.09)	-3.89 (-18.33, 10.54)	-3.39 (-18.07, 11.29)	-12.86 (-37.78, 12.06)
PUFA*B12*FA.high	-16.43 (-40.71, 7.84)	-13.95 (-38.67, 10.78)	-13.49 (-38.62, 11.64)	-4.51 (-18.68, 9.67)	-3.73 (-18.10, 10.63)	-16.43 (-40.71, 7.84)
**Visuospatial Constrtuction**						
PUFA	6.28 (-8.17, 20.73)	4.88 (-9.99, 19.74)	5.28 (-9.86, 20.41)	**-11.14 (-21.33, -0.94)***	**-11.82 (-22.25, -1.39)***	**-12.20 (-22.86, -1.54)***
B12	2.74 (-18.36, 23.84)	3.52 (-17.77, 24.81)	3.53 (-18.11, 25.17)	5.18 (-4.69, 15.05)	4.38 (-6.28, 15.03)	3.93 (-6.93, 14.79)
FA.medium	-5.45 (-16.39, 5.49)	-6.34 (-17.64, 4.97)	-6.49 (-17.97, 4.98)	6.88 (-0.66, 14.42)	6.65 (-1.15, 14.46)	6.94 (-1.06, 14.93)
FA.high	-3.05 (-13.85, 7.75)	-3.42 (-14.40, 7.56)	-3.43 (-14.67, 7.82)	7.63 (-0.96, 16.23)	7.61 (-1.30, 16.53)	7.84 (-1.21, 16.88)
PUFA*B12	3.95 (-22.99, 30.89)	2.92 (-24.27, 30.10)	2.92 (-24.86, 30.70)	-3.98 (-19.27, 11.31)	-5.05 (-20.76, 10.66)	-5.88 (-21.99, 10.22)
PUFA*FA.medium	-5.82 (-21.61, 9.97)	-4.33 (-20.70, 12.04)	-5.03 (-21.76, 11.71)	10.11 (-1.67, 21.89)	10.58 (-1.49, 22.66)	10.93 (-1.36, 23.22)
PUFA*FA.high	-6.67 (-21.85, 8.52)	-4.75 (-20.45, 10.96)	-5.28 (-21.29, 10.73)	**12.19 (1.30, 23.08)***	**12.66 (1.57, 23.76)***	**13.19 (1.80, 24.58)***
B12*FA.medium	-7.15 (-29.46, 15.17)	-8.73 (-31.23, 13.78)	-8.71 (-31.63, 14.20)	-4.14 (-15.45, 7.16)	-3.02 (-15.26, 9.22)	-2.81 (-15.23, 9.61)
B12*FA.high	-2.32 (-23.58, 18.94)	-3.01 (-24.42, 18.41)	-2.94 (-24.71, 18.82)	4.98 (-7.59, 17.55)	6.19 (-7.12, 19.49)	6.79 (-6.80, 20.37)
PUFA*B12*FA.medium	-7.72 (-35.56, 20.12)	-6.37 (-34.45, 21.72)	-6.40 (-34.98, 22.18)	5.15 (-11.12, 21.43)	5.71 (-11.06, 22.48)	6.50 (-10.64, 23.64)
PUFA*B12*FA.high	-3.91 (-31.03, 23.21)	-3.04 (-30.41, 24.33)	-2.99 (-30.99, 25.02)	0.18 (-15.83, 16.18)	1.12 (-15.34, 17.59)	1.75 (-15.03, 18.52)
**Total RBANS**						
PUFA	10.03 (-1.03, 21.09)	8.16 (-3.26, 19.59)	8.38 (-3.18, 19.94)	**-10.51 (-18.64, -2.38)***	**-11.10 (-19.27, -2.92)***	**-11.72 (-20.05, -3.39)***
B12	8.94 (-7.21, 25.09)	8.95 (-7.41, 25.31)	8.88 (-7.64, 25.41)	5.12 (-2.76, 12.99)	2.04 (-6.31, 10.39)	1.68 (-6.81, 10.17)
FA.medium	-4.66 (-13.03, 3.72)	-4.62 (-13.31, 4.07)	-5.09 (-13.85, 3.68)	2.84 (-3.18, 8.85)	3.94 (-2.17, 10.06)	4.48 (-1.76, 10.73)
FA.high	-0.82 (-9.08, 7.44)	-0.51 (-8.95, 7.93)	-1.20 (-9.79, 7.39)	2.53 (-4.33, 9.39)	3.63 (-3.36, 10.61)	3.96 (-3.11, 11.03)
PUFA*B12	15.29 (-5.32, 35.91)	13.44 (-7.46, 34.33)	12.06 (-9.15, 33.27)	-6.15 (-18.34, 6.05)	-8.66 (-20.97, 3.65)	-9.44 (-22.02, 3.15)
PUFA*FA.medium	-9.62 (-21.70, 2.47)	-7.36 (-19.94, 5.22)	-8.27 (-21.05, 4.51)	**11.64 (2.24, 21.03)***	**13.27 (3.81, 22.73)***	**13.86 (4.26, 23.46)***
PUFA*FA.high	-8.67 (-20.29, 2.95)	-6.67 (-18.74, 5.40)	-7.33 (-19.55, 4.90)	**10.01 (1.32, 18.70)***	**10.29 (1.60, 18.98)***	**11.09 (2.20, 19.99)***
B12*FA.medium	-12.33 (-29.41, 4.75)	-12.67 (-29.97, 4.62)	-12.31 (-29.80, 5.19)	-7.38 (-16.40, 1.64)	-4.46 (-14.05, 5.13)	-3.96 (-13.66, 5.74)
B12*FA.high	-10.11 (-26.38, 6.16)	-10.55 (-27.01, 5.91)	-10.33 (-26.95, 6.29)	0.03 (-10.01, 10.06)	3.34 (-7.09, 13.76)	3.82 (-6.79, 14.44)
PUFA*B12*FA.medium	-18.52 (-39.83, 2.78)	-16.29 (-37.88, 5.29)	-15.31 (-37.14, 6.51)	8.31 (-4.67, 21.30)	10.51 (-2.63, 23.65)	11.04 (-2.36, 24.43)
PUFA*B12*FA.high	-16.61 (-37.36, 4.15)	-14.68 (-35.72, 6.36)	-13.12 (-34.50, 8.27)	6.57 (-6.20, 19.34)	9.01 (-3.89, 21.92)	9.69 (-3.42, 22.79)

Results with p<0.05 are indicated in bold with *

Table S4. The results of the sex-stratified linear regression models on RBANS total and subdomain scores with UFA:SFA substituted for PUFA.

	Female			Male		
	**Model 1**	**Model 2**	**Model 3**	**Model 1**	**Model 2**	**Model 3**
**RBANS domain**			**β (95% CI)**			
**Attention**						
UFA:SFA	-2.20 (-7.22, 2.81)	-2.40 (-7.54, 2.73)	-2.41 (-7.58, 2.76)	-3.99 (-14.25, 6.27)	-4.15 (-14.63, 6.32)	-3.14 (-13.62, 7.33)
B12	-1.81 (-10.95, 7.34)	-3.64 (-13.43, 6.15)	-2.77 (-12.98, 7.44)	5.19 (-6.40, 16.78)	4.21 (-7.61, 16.03)	3.15 (-8.78, 15.08)
FA.medium	-2.06 (-9.88, 5.75)	-2.14 (-10.39, 6.12)	-3.00 (-11.44, 5.44)	-8.32 (-16.98, 0.35)	-8.85 (-17.67, -0.02)	**-9.01 (-17.76, -0.26)***
FA.high	-2.50 (-10.52, 5.52)	-1.31 (-9.59, 6.96)	-2.80 (-11.28, 5.68)	-2.84 (-11.69, 6.02)	-3.03 (-12.02, 5.96)	-2.93 (-11.90, 6.04)
UFA:SFA *B12	0.81 (-6.49, 8.11)	-0.71 (-8.27, 6.85)	-0.45 (-8.07, 7.16)	-12.80 (-28.14, 2.53)	-13.65 (-29.25, 1.96)	-13.73 (-29.17, 1.72)
UFA:SFA*FA.medium	1.89 (-5.07, 8.84)	1.56 (-5.52, 8.65)	1.17 (-5.98, 8.33)	-0.28 (-11.67, 11.10)	-0.15 (-11.84, 11.53)	-0.96 (-12.58, 10.66)
UFA:SFA *FA.high	-0.46 (-7.32, 6.40)	-1.19 (-8.17, 5.80)	-1.67 (-8.67, 5.34)	4.60 (-6.27, 15.47)	4.77 (-6.30, 15.84)	3.80 (-7.23, 14.83)
B12*FA.medium	0.32 (-11.46, 12.10)	2.13 (-10.55, 14.80)	0.82 (-12.51, 14.14)	-6.27 (-19.06, 6.52)	-5.20 (-18.32, 7.91)	-4.82 (-18.10, 8.46)
B12*FA.high	5.00 (-6.15, 16.16)	6.65 (-5.11, 18.40)	6.18 (-5.86, 18.23)	-5.88 (-17.86, 6.10)	-4.92 (-17.13, 7.29)	-4.10 (-16.37, 8.18)
UFA:SFA*B12*FA.medium	-1.58 (-10.26, 7.11)	0.33 (-8.64, 9.29)	0.30 (-8.73, 9.33)	13.77 (-2.72, 30.26)	15.49 (-1.42, 32.39)	16.78 (-0.05, 33.60)
UFA:SFA*B12*FA.high	2.26 (-6.34, 10.86)	5.02 (-4.12, 14.16)	5.45 (-3.75, 14.65)	12.58 (-2.91, 28.07)	13.35 (-2.44, 29.14)	13.09 (-2.55, 28.73)
**Delayed memory**						
UFA:SFA	**-4.35 (-7.69, -1.02)***	**-4.33 (-7.73, -0.94)***	**-4.54 (-7.99, -1.10)***	4.12 (-3.71, 11.96)	3.67 (-4.36, 11.69)	3.24 (-4.89, 11.37)
B12	7.09 (1.00, 13.17)	6.42 (-0.05, 12.90)	7.37 (0.56, 14.17)	1.46 (-7.39, 10.31)	1.85 (-7.20, 10.90)	2.81 (-6.45, 12.06)
FA.medium	-3.19 (-8.40, 2.01)	-3.83 (-9.29, 1.64)	-4.36 (-9.99, 1.26)	-0.58 (-7.19, 6.04)	-0.89 (-7.65, 5.86)	-1.22 (-8.01, 5.57)
FA.high	-2.37 (-7.70, 2.96)	-2.71 (-8.18, 2.77)	-3.18 (-8.84, 2.47)	3.46 (-3.31, 10.22)	3.30 (-3.58, 10.19)	2.83 (-4.13, 9.79)
UFA:SFA *B12	-4.17 (-9.02, 0.69)	-4.94 (-9.94, 0.06)	-5.21 (-10.28, -0.13)	9.24 (-2.47, 20.95)	8.64 (-3.31, 20.59)	8.51 (-3.48, 20.50)
UFA:SFA*FA.medium	1.37 (-3.26, 5.99)	1.37 (-3.31, 6.06)	1.62 (-3.15, 6.38)	-5.98 (-14.68, 2.72)	-5.63 (-14.58, 3.32)	-5.46 (-14.48, 3.56)
UFA:SFA *FA.high	4.12 (-0.44, 8.68)	4.02 (-0.60, 8.64)	3.96 (-0.71, 8.62)	-4.24 (-12.54, 4.07)	-3.86 (-12.33, 4.62)	-3.58 (-12.14, 4.98)
B12*FA.medium	**-9.99 (-17.83, -2.16)***	**-8.76 (-17.14, -0.37)***	**-9.87 (-18.75, -0.99)***	1.24 (-8.53, 11.01)	0.38 (-9.67, 10.42)	-0.73 (-11.03, 9.58)
B12*FA.high	**-9.31 (-16.73, -1.90)***	**-8.06 (-15.83, -0.28)***	**-8.85 (-16.88, -0.82)***	-2.66 (-11.81, 6.49)	-3.13 (-12.48, 6.22)	-4.09 (-13.62, 5.43)
UFA:SFA*B12*FA.medium	2.43 (-3.34, 8.21)	2.80 (-3.13, 8.73)	3.07 (-2.95, 9.09)	-7.86 (-20.45, 4.73)	-6.60 (-19.55, 6.35)	-6.27 (-19.32, 6.79)
UFA:SFA*B12*FA.high	6.09 (0.37, 11.81)	7.46 (1.42, 13.51)	7.85 (1.71, 13.98)	-9.55 (-21.38, 2.27)	-9.12 (-21.22, 2.97)	-9.04 (-21.18, 3.09)
**Immediate memory**						
UFA:SFA	-2.13 (-5.87, 1.62)	-2.65 (-6.28, 0.98)	-2.69 (-6.38, 0.99)	2.51 (-6.06, 11.09)	1.90 (-6.86, 10.67)	-2.13 (-5.87, 1.62)
B12	2.51 (-4.31, 9.34)	2.75 (-4.17, 9.68)	3.27 (-4.01, 10.56)	-2.24 (-11.93, 7.45)	-2.30 (-12.19, 7.59)	2.51 (-4.31, 9.34)
FA.medium	-0.94 (-6.77, 4.89)	-2.09 (-7.93, 3.74)	-2.51 (-8.53, 3.51)	4.23 (-3.01, 11.47)	4.26 (-3.12, 11.64)	-0.94 (-6.77, 4.89)
FA.high	-0.59 (-6.57, 5.38)	-1.11 (-6.96, 4.74)	-1.70 (-7.76, 4.35)	5.13 (-2.27, 12.52)	5.19 (-2.33, 12.71)	-0.59 (-6.57, 5.38)
UFA:SFA *B12	-1.53 (-6.97, 3.91)	-2.54 (-7.88, 2.81)	-2.51 (-7.95, 2.92)	1.44 (-11.37, 14.26)	0.90 (-12.15, 13.96)	-1.53 (-6.97, 3.91)
UFA:SFA*FA.medium	1.18 (-4.01, 6.37)	1.41 (-3.60, 6.41)	1.34 (-3.77, 6.44)	-4.76 (-14.27, 4.76)	-3.96 (-13.74, 5.82)	1.18 (-4.01, 6.37)
UFA:SFA *FA.high	-0.12 (-5.23, 5.00)	0.27 (-4.67, 5.21)	0.09 (-4.90, 5.09)	-1.29 (-10.38, 7.79)	-0.66 (-9.93, 8.60)	-0.12 (-5.23, 5.00)
B12*FA.medium	**-9.01 (-17.80, -0.23)**	**-9.63 (-18.59, -0.67)**	**-10.37 (-19.87, -0.87)**	4.50 (-6.19, 15.19)	4.14 (-6.84, 15.11)	**-9.01 (-17.80, -0.23)***
B12*FA.high	-0.23 (-8.55, 8.09)	0.42 (-7.89, 8.74)	0.07 (-8.53, 8.66)	2.48 (-7.53, 12.49)	2.13 (-8.09, 12.34)	-0.23 (-8.55, 8.09)
UFA:SFA*B12*FA.medium	-1.38 (-7.86, 5.10)	-1.45 (-7.79, 4.89)	-1.38 (-7.82, 5.06)	-1.14 (-14.92, 12.64)	0.01 (-14.13, 14.16)	-1.38 (-7.86, 5.10)
UFA:SFA*B12*FA.high	4.04 (-2.37, 10.46)	5.28 (-1.18, 11.74)	5.49 (-1.08, 12.05)	-1.41 (-14.35, 11.54)	-0.98 (-14.19, 12.23)	4.04 (-2.37, 10.46)
**Language**						
UFA:SFA	-3.73 (-7.54, 0.07)	-3.80 (-7.67, 0.06)	-3.97 (-7.89, -0.04)	1.66 (-7.25, 10.57)	0.56 (-8.50, 9.62)	0.46 (-8.76, 9.68)
B12	4.78 (-2.16, 11.73)	4.04 (-3.34, 11.41)	4.41 (-3.34, 12.16)	0.06 (-10.00, 10.13)	1.04 (-9.18, 11.27)	0.89 (-9.60, 11.38)
FA.medium	0.01 (-5.92, 5.94)	1.42 (-4.80, 7.64)	1.24 (-5.16, 7.65)	-1.39 (-8.91, 6.14)	-1.92 (-9.55, 5.70)	-1.72 (-9.41, 5.98)
FA.high	0.41 (-5.67, 6.49)	1.36 (-4.87, 7.60)	1.16 (-5.28, 7.60)	-1.13 (-8.82, 6.56)	-2.25 (-10.02, 5.52)	-1.97 (-9.87, 5.92)
UFA:SFA *B12	**5.89 (0.36, 11.43)***	5.56 (-0.13, 11.26)	5.36 (-0.42, 11.14)	5.96 (-7.36, 19.27)	4.39 (-9.09, 17.89)	4.43 (-9.16, 18.02)
UFA:SFA*FA.medium	**5.61 (0.33, 10.88)***	4.99 (-0.35, 10.33)	5.12 (-0.31, 10.55)	-3.86 (-13.75, 6.03)	-3.08 (-13.19, 7.02)	-2.89 (-13.11, 7.33)
UFA:SFA *FA.high	2.83 (-2.37, 8.03)	2.71 (-2.55, 7.97)	2.71 (-2.60, 8.03)	0.24 (-9.19, 9.69)	1.19 (-8.38, 10.76)	1.35 (-8.35, 11.06)

B12*FA.medium	**-10.78 (-19.72, -1.85)**	**-10.46 (-20.01, -0.92)**	**-10.54 (-20.65, -0.44)**	-0.65 (-11.76, 10.46)	-1.69 (-13.04, 9.65)	-1.47 (-13.15, 10.21)
B12*FA.high	-2.97 (-11.43, 5.50)	-2.37 (-11.23, 6.48)	-2.59 (-11.74, 6.54)	-0.39 (-10.79, 10.02)	-1.55 (-12.10, 9.00)	-1.32 (-12.12, 9.47)
UFA:SFA*B12*FA.medium	-2.25 (-8.84, 4.33)	-1.82 (-8.58, 4.93)	-1.82 (-8.67, 5.04)	-6.26 (-20.58, 8.06)	-3.66 (-18.27, 10.96)	-3.94 (-18.73, 10.86)
UFA:SFA*B12*FA.high	-6.39 (-12.92, 0.13)	-6.39 (-13.27, 0.49)	-5.96 (-12.94, 1.02)	-6.75 (-20.20, 6.70)	-5.21 (-18.86, 8.45)	-5.13 (-18.89, 8.62)
**Visuospatial construction**						
UFA:SFA	-3.06 (-7.62, 1.50)	-3.45 (-8.09, 1.19)	-3.83 (-8.49, 0.84)	-0.89 (-10.75, 8.95)	-2.19 (-12.06, 7.67)	-3.20 (-13.15, 6.75)
B12	5.22 (-3.09, 13.54)	4.42 (-4.42, 13.26)	5.15 (-4.07, 14.37)	0.64 (-10.49, 11.77)	2.82 (-8.31, 13.96)	4.79 (-6.54, 16.12)
FA.medium	3.16 (-3.95, 10.27)	1.86 (-5.59, 9.32)	1.67 (-5.94, 9.29)	-3.36 (-11.68, 4.96)	-5.01 (-13.32, 3.30)	-5.54 (-13.84, 2.77)
FA.high	2.19 (-5.10, 9.48)	2.12 (-5.35, 9.61)	2.18 (-5.48, 9.84)	2.47 (-6.02, 10.97)	1.06 (-7.41, 9.53)	0.13 (-8.39, 8.65)
UFA:SFA *B12	-2.45 (-9.08, 4.18)	-4.01 (-10.84, 2.82)	-4.59 (-11.47, 2.29)	-2.94 (-17.66, 11.78)	-3.02 (-17.72, 11.68)	-3.17 (-17.84, 11.49)
UFA:SFA*FA.medium	3.19 (-3.13, 9.51)	3.05 (-3.34, 9.45)	3.56 (-2.89, 10.03)	-2.09 (-13.03, 8.83)	-2.16 (-13.18, 8.84)	-1.69 (-12.73, 9.34)
UFA:SFA *FA.high	1.12 (-5.11, 7.36)	0.91 (-5.39, 7.23)	1.09 (-5.23, 7.42)	1.27 (-9.16, 11.71)	2.55 (-7.87, 12.98)	3.28 (-7.19, 13.76)
B12*FA.medium	-4.45 (-15.15, 6.26)	-3.58 (-15.03, 7.87)	-3.72 (-15.75, 8.32)	-2.00 (-14.29, 10.27)	-4.91 (-17.27, 7.44)	-6.76 (-19.37, 5.85)
B12*FA.high	-4.47 (-14.61, 5.67)	-3.11 (-13.74, 7.51)	-3.69 (-14.57, 7.20)	0.96 (-10.54, 12.46)	-0.70 (-12.21, 10.79)	-2.61 (-14.27, 9.04)
UFA:SFA*B12*FA.medium	3.68 (-4.22, 11.57)	5.01 (-3.09, 13.11)	5.17 (-2.98, 13.33)	1.73 (-14.09, 17.56)	3.09 (-12.83, 19.03)	3.08 (-12.89, 19.06)
UFA:SFA*B12*FA.high	1.26 (-6.56, 9.08)	4.09 (-4.16, 12.35)	4.81 (-3.49, 13.13)	3.85 (-11.01, 18.72)	3.62 (-11.26, 18.50)	3.80 (-11.04, 18.65)
**Total RBANS**						
UFA:SFA	**-5.05 (-8.65, -1.45)**	**-5.34 (-8.96, -1.71)**	0.95 (-6.63, 8.54)	-0.17 (-7.83, 7.50)	-0.43 (-8.17, 7.31)	-5.05 (-8.65, -1.45)
B12	4.42 (-2.45, 11.28)	5.67 (-1.50, 12.84)	1.47 (-7.10, 10.04)	2.25 (-6.40, 10.90)	3.01 (-5.80, 11.83)	4.42 (-2.45, 11.28)
FA.medium	-1.31 (-7.10, 4.48)	-2.08 (-8.00, 3.84)	-2.71 (-9.12, 3.70)	-3.51 (-9.96, 2.94)	-3.85 (-10.31, 2.62)	-1.31 (-7.10, 4.48)
FA.high	-0.21 (-6.01, 5.59)	-1.06 (-7.02, 4.89)	2.71 (-3.83, 9.26)	1.93 (-4.65, 8.50)	1.50 (-5.13, 8.13)	-0.21 (-6.01, 5.59)
UFA:SFA *B12	-2.08 (-7.38, 3.22)	-2.38 (-7.73, 2.96)	0.16 (-11.17, 11.50)	-0.91 (-12.32, 10.51)	-1.05 (-12.46, 10.36)	-2.08 (-7.38, 3.22)
UFA:SFA*FA.medium	3.51 (-1.46, 8.48)	3.73 (-1.29, 8.75)	-4.16 (-12.58, 4.26)	-3.45 (-12.00, 5.10)	-3.41 (-11.99, 5.18)	3.51 (-1.46, 8.48)
UFA:SFA *FA.high	2.08 (-2.81, 6.98)	1.93 (-2.98, 6.85)	0.21 (-7.83, 8.25)	1.25 (-6.85, 9.34)	1.37 (-6.78, 9.52)	2.08 (-2.81, 6.98)
B12*FA.medium	**-9.66 (-18.55, -0.78)**	**-11.00 (-20.35, -1.66)**	-0.97 (-10.42, 8.50)	-2.24 (-11.84, 7.36)	-3.28 (-13.09, 6.53)	**-9.66 (-18.55, -0.78)**
B12*FA.high	-2.35 (-10.60, 5.89)	-3.30 (-11.76, 5.15)	-1.97 (-10.83, 6.90)	-2.82 (-11.75, 6.11)	-3.63 (-12.69, 5.44)	-2.35 (-10.60, 5.89)
UFA:SFA*B12*FA.medium	1.65 (-4.63, 7.94)	1.91 (-4.43, 8.25)	-0.48 (-12.67, 11.71)	1.81 (-10.56, 14.18)	2.36 (-10.07, 14.79)	1.65 (-4.63, 7.94)
UFA:SFA*B12*FA.high	4.79 (-1.62, 11.19)	5.46 (-1.00, 11.92)	-0.36 (-11.81, 11.09)	0.54 (-11.02, 12.09)	0.59 (-10.97, 12.14)	4.79 (-1.62, 11.19)

Results with p<0.05 are indicated in bold

Table S5. The results of the amyloid-stratified-stratified linear regression models on RBANS total and subdomain scores with UFA:SFA substituted for PUFA.

	**Positive**			**Negative**		
	**Model 1**	**Model 2**	**Model 3**	**Model 1**	**Model 2**	**Model 3**
**RBANS domain**			**β (95% CI)**			
**Attention**						
UFA:SFA	-4.73 (-11.15, 1.70)	-4.97 (-11.71, 1.78)	-5.44 (-12.07, 1.19)	1.18 (-5.01, 7.38)	0.55 (-5.71, 6.80)	0.23 (-6.09, 6.56)
B12	-0.98 (-12.29, 10.34)	0.61 (-11.18, 12.41)	4.48 (-7.31, 16.28)	2.08 (-7.41, 11.57)	-0.31 (-10.25, 9.64)	-0.05 (-10.10, 10.01)
FA.medium	-4.99 (-13.29, 3.31)	-6.41 (-15.13, 2.31)	-7.91 (-16.47, 0.65)	-1.46 (-9.30, 6.38)	-0.66 (-8.70, 7.38)	-1.04 (-9.21, 7.14)
FA.high	0.92 (-6.97, 8.81)	0.22 (-7.98, 8.42)	-2.82 (-11.05, 5.41)	-1.20 (-9.87, 7.48)	0.43 (-8.28, 9.14)	0.13 (-8.71, 8.96)
UFA:SFA *B12	-2.80 (-10.81, 5.20)	-3.12 (-11.56, 5.33)	-3.78 (-12.05, 4.49)	-1.93 (-11.99, 8.14)	-3.66 (-14.03, 6.70)	-4.28 (-14.79, 6.23)
UFA:SFA*FA.medium	3.49 (-4.34, 11.32)	3.66 (-4.42, 11.75)	3.10 (-4.85, 11.05)	-2.39 (-10.61, 5.83)	-2.69 (-10.89, 5.50)	-1.85 (-10.32, 6.62)
UFA:SFA *FA.high	6.50 (-1.08, 14.08)	6.94 (-0.93, 14.81)	7.07 (-0.67, 14.81)	-3.74 (-11.25, 3.77)	-3.33 (-10.93, 4.27)	-2.92 (-10.63, 4.78)
B12*FA.medium	0.40 (-13.38, 14.19)	-0.58 (-14.88, 13.72)	-4.43 (-18.67, 9.81)	-5.01 (-16.40, 6.38)	-2.16 (-14.23, 9.91)	-2.39 (-14.65, 9.86)
B12*FA.high	0.56 (-11.22, 12.33)	-1.86 (-14.14, 10.41)	-5.92 (-18.20, 6.35)	-3.11 (-15.41, 9.18)	-1.08 (-13.82, 11.67)	-1.03 (-13.88, 11.82)
UFA:SFA*B12*FA.medium	2.83 (-6.61, 12.27)	3.31 (-6.74, 13.36)	4.08 (-5.78, 13.95)	0.51 (-11.26, 12.29)	3.68 (-8.26, 15.62)	3.97 (-8.12, 16.06)
UFA:SFA*B12*FA.high	2.52 (-5.84, 10.87)	2.84 (-6.03, 11.71)	3.21 (-5.47, 11.89)	4.62 (-6.36, 15.60)	6.52 (-4.83, 17.86)	7.11 (-4.37, 18.60)
**Delayed memory**						
UFA:SFA	-1.05 (-6.14, 4.04)	-1.90 (-7.32, 3.51)	-1.41 (-6.82, 4.01)	**-4.63 (-8.65, -0.61)**	**-4.16 (-8.24, -0.07)**	**-3.78 (-7.86, 0.298)**

B12	4.52 (-4.45, 13.48)	6.26 (-3.21, 15.73)	6.56 (-3.07, 16.19)	5.70 (-0.45, 11.86)	4.65 (-1.85, 11.14)	4.47 (-2.01, 10.95)
FA.medium	-1.05 (-7.63, 5.52)	-2.13 (-9.13, 4.87)	-2.26 (-9.25, 4.73)	-4.41 (-9.49, 0.68)	-3.59 (-8.85, 1.66)	-3.28 (-8.55, 1.996)
FA.high	0.24 (-6.00, 6.50)	-0.76 (-7.34, 5.82)	-0.79 (-7.52, 5.93)	-2.78 (-8.41, 2.85)	-2.71 (-8.40, 2.98)	-2.52 (-8.22, 3.18)
UFA:SFA *B12	-3.93 (-10.27, 2.41)	-4.58 (-11.36, 2.20)	-4.81 (-11.57, 1.94)	0.53 (-6.01, 7.06)	-0.35 (-7.12, 6.42)	0.45 (-6.33, 7.23)
UFA:SFA*FA.medium	-1.51 (-7.72, 4.69)	-0.85 (-7.35, 5.64)	-1.55 (-8.05, 4.94)	1.39 (-3.94, 6.73)	1.44 (-3.92, 6.79)	0.21 (-5.25, 5.67)
UFA:SFA *FA.high	0.82 (-5.19, 6.82)	1.79 (-4.53, 8.11)	1.11 (-5.21, 7.43)	**5.05 (0.18, 9.93)**	4.81 (-0.15, 9.77)	4.23 (-0.74, 9.19)
B12*FA.medium	-4.93 (-15.86, 5.99)	-6.79 (-18.28, 4.69)	-6.80 (-18.43, 4.82)	-3.37 (-10.77, 4.02)	-2.43 (-10.31, 5.46)	-1.85 (-9.75, 6.06)
B12*FA.high	-7.17 (-16.50, 2.16)	-9.08 (-18.94, 0.77)	-9.45 (-19.48, 0.57)	-1.77 (-9.75, 6.22)	-0.18 (-8.50, 8.14)	-0.37 (-8.66, 7.92)
UFA:SFA*B12*FA.medium	0.96 (-6.51, 8.44)	1.74 (-6.33, 9.81)	1.89 (-6.16, 9.96)	2.65 (-4.99, 10.29)	3.31 (-4.49, 11.10)	2.65 (-5.15, 10.44)
UFA:SFA*B12*FA.high	2.88 (-3.74, 9.49)	3.47 (-3.65, 10.60)	3.59 (-3.49, 10.68)	-1.12 (-8.24, 6.01)	-0.61 (-8.02, 6.79)	-1.27 (-8.68, 6.14)
**Immediate memory**						
UFA:SFA	-2.40 (-7.98, 3.18)	-2.17 (-8.07, 3.73)	-2.48 (-8.45, 3.49)	-0.39 (-4.84, 4.05)	-1.17 (-5.63, 3.29)	-0.90 (-5.37, 3.57)
B12	0.92 (-8.90, 10.74)	0.88 (-9.44, 11.20)	2.39 (-8.22, 13.01)	1.46 (-5.34, 8.26)	2.36 (-4.73, 9.45)	1.91 (-5.19, 9.01)
FA.medium	6.08 (-1.13, 13.28)	6.32 (-1.31, 13.94)	5.78 (-1.93, 13.49)	-0.11 (-5.74, 5.51)	-0.78 (-6.51, 4.95)	-0.10 (-5.88, 5.67)
FA.high	4.92 (-1.93, 11.78)	4.93 (-2.25, 12.10)	3.92 (-3.49, 11.33)	-2.09 (-8.31, 4.13)	-2.22 (-8.43, 3.99)	-1.63 (-7.87, 4.62)
UFA:SFA *B12	-1.50 (-8.45, 5.45)	-1.12 (-8.50, 6.27)	-1.45 (-8.90, 5.99)	-1.04 (-8.26, 6.18)	-1.06 (-8.45, 6.32)	-0.73 (-8.15, 6.69)
UFA:SFA*FA.medium	-1.80 (-8.60, 5.00)	-1.73 (-8.80, 5.35)	-1.78 (-8.94, 5.37)	-0.99 (-6.89, 4.90)	-1.30 (-7.15, 4.54)	-2.36 (-8.34, 3.63)
UFA:SFA *FA.high	4.37 (-2.21, 10.95)	4.06 (-2.83, 10.95)	4.29 (-2.68, 11.26)	-0.75 (-6.14, 4.63)	0.54 (-4.87, 5.96)	0.39 (-5.05, 5.84)
B12*FA.medium	2.26 (-9.71, 14.23)	2.77 (-9.74, 15.28)	1.07 (-11.75, 13.89)	-2.94 (-11.11, 5.23)	-4.62 (-13.22, 4.00)	-3.82 (-12.48, 4.84)
B12*FA.high	-1.72 (-11.95, 8.50)	-2.09 (-12.83, 8.66)	-3.64 (-14.69, 7.41)	4.95 (-3.87, 13.76)	3.51 (-5.58, 12.60)	3.69 (-5.39, 12.76)
UFA:SFA*B12*FA.medium	-5.42 (-13.62, 2.77)	-6.09 (-14.89, 2.70)	-5.61 (-14.50, 3.27)	2.93 (-5.51, 11.37)	3.35 (-5.16, 11.86)	3.07 (-5.47, 11.61)
UFA:SFA*B12*FA.high	0.81 (-6.44, 8.06)	0.65 (-7.11, 8.42)	0.89 (-6.92, 8.70)	1.15 (-6.73, 9.02)	0.87 (-7.22, 8.96)	0.63 (-7.48, 8.75)
**Language**						
UFA:SFA	-3.35 (-9.06, 2.35)	-5.39 (-11.37, 0.59)	-5.05 (-11.11, 1.01)	-2.54 (-7.20, 2.13)	-2.49 (-7.19, 2.21)	-3.35 (-9.06, 2.35)
B12	5.39 (-4.65, 15.44)	8.49 (-1.97, 18.94)	7.49 (-3.29, 18.27)	4.56 (-2.86, 11.98)	4.26 (-3.21, 11.72)	5.39 (-4.65, 15.44)
FA.medium	0.94 (-6.42, 8.31)	-1.21 (-8.94, 6.51)	-0.89 (-8.72, 6.94)	-1.08 (-7.08, 4.92)	-0.64 (-6.71, 5.43)	0.94 (-6.42, 8.31)
FA.high	4.16 (-2.84, 11.17)	2.21 (-5.06, 9.48)	2.81 (-4.72, 10.33)	-4.89 (-11.39, 1.61)	-4.47 (-11.03, 2.09)	4.16 (-2.84, 11.17)
UFA:SFA *B12	3.41 (-3.69, 10.52)	1.39 (-6.09, 8.88)	1.63 (-5.93, 9.19)	**8.57 (0.84, 16.31)**	**8.45 (0.64, 16.25)**	3.41 (-3.69, 10.52)
UFA:SFA*FA.medium	2.21 (-4.74, 9.16)	3.83 (-3.34, 11.00)	3.69 (-3.57, 10.96)	3.19 (-2.93, 9.30)	2.72 (-3.57, 9.01)	2.21 (-4.74, 9.16)
UFA:SFA *FA.high	5.37 (-1.36, 12.10)	**7.56 (0.59, 14.54)**	**7.23 (0.15, 14.31)**	3.18 (-2.49, 8.85)	3.35 (-2.38, 9.07)	5.37 (-1.36, 12.10)
B12*FA.medium	-6.09 (-18.34, 6.14)	-10.00 (-22.68, 2.67)	-8.74 (-21.76, 4.28)	**-10.20 (-19.21, -1.19)**	**-9.54 (-18.64, -0.44)**	-6.09 (-18.34, 6.14)
B12*FA.high	-7.17 (-17.62, 3.28)	-10.27 (-21.15, 0.61)	-9.27 (-20.49, 1.94)	-4.16 (-13.68, 5.35)	-3.93 (-13.47, 5.62)	-7.17 (-17.62, 3.28)
UFA:SFA*B12*FA.medium	-0.82 (-9.19, 7.56)	1.68 (-7.23, 10.59)	1.28 (-7.74, 10.30)	**-10.59 (-19.50, -1.69)**	**-10.65 (-19.62, -1.67)**	-0.82 (-9.19, 7.56)
UFA:SFA*B12*FA.high	-5.54 (-12.95, 1.88)	-3.65 (-11.52, 4.21)	-3.84 (-11.78, 4.09)	-7.00 (-15.47, 1.46)	-6.79 (-15.32, 1.74)	-5.54 (-12.95, 1.88)
**Visuospatial construction**						
UFA:SFA	0.16 (-6.36, 6.68)	-1.01 (-7.83, 5.81)	-1.10 (-8.03, 5.82)	-4.77 (-9.99, 0.45)	-4.74 (-10.12, 0.64)	-4.72 (-10.18, 0.74)
B12	1.87 (-9.62, 13.35)	4.10 (-7.82, 16.03)	4.11 (-8.21, 16.42)	5.79 (-2.21, 13.78)	5.61 (-2.94, 14.16)	5.59 (-3.09, 14.26)
FA.medium	-4.50 (-12.92, 3.92)	-6.85 (-15.67, 1.95)	-6.78 (-15.72, 2.17)	-0.17 (-6.77, 6.44)	-0.76 (-7.68, 6.14)	-0.73 (-7.79, 6.32)
FA.high	-0.09 (-8.11, 7.91)	-1.70 (-9.99, 6.58)	-1.37 (-9.96, 7.23)	1.78 (-5.52, 9.08)	1.41 (-6.07, 8.90)	1.44 (-6.19, 9.07)
UFA:SFA *B12	-1.25 (-9.37, 6.88)	-2.11 (-10.65, 6.42)	-2.28 (-10.91, 6.36)	-4.74 (-13.22, 3.74)	-4.94 (-13.85, 3.96)	-4.92 (-13.99, 4.15)
UFA:SFA*FA.medium	2.09 (-5.86, 10.04)	2.79 (-5.38, 10.97)	2.92 (-5.39, 11.22)	1.49 (-5.43, 8.42)	1.24 (-5.81, 8.29)	1.19 (-6.12, 8.50)
UFA:SFA *FA.high	0.52 (-7.18, 8.21)	1.80 (-6.15, 9.76)	1.94 (-6.15, 10.02)	3.15 (-3.17, 9.48)	3.11 (-3.41, 9.65)	3.10 (-3.55, 9.75)
B12*FA.medium	-9.49 (-23.49, 4.50)	-12.60 (-27.05, 1.85)	-12.86 (-27.73, 2.01)	-4.63 (-14.22, 4.96)	-4.34 (-14.72, 6.03)	-4.32 (-14.90, 6.25)
B12*FA.high	-1.33 (-13.29, 10.62)	-3.43 (-15.83, 8.98)	-3.39 (-16.20, 9.43)	2.77 (-7.59, 13.12)	3.07 (-7.88, 14.03)	3.08 (-8.02, 14.17)
UFA:SFA*B12*FA.medium	4.79 (-4.78, 14.38)	5.97 (-4.18, 16.13)	6.28 (-4.02, 16.59)	3.39 (-6.52, 13.31)	3.78 (-6.47, 14.05)	3.78 (-6.65, 14.21)
UFA:SFA*B12*FA.high	1.32 (-7.16, 9.80)	2.28 (-6.68, 11.25)	2.45 (-6.61, 11.52)	1.89 (-7.35, 11.15)	2.03 (-7.71, 11.79)	2.01 (-7.90, 11.92)
**Total RBANS**						
UFA:SFA	-3.20 (-8.21, 1.81)	-4.43 (-9.66, 0.79)	-4.47 (-9.73, 0.80)	-3.46 (-7.69, 0.76)	-3.73 (-8.01, 0.56)	-3.66 (-8.00, 0.68)
B12	2.76 (-6.06, 11.58)	5.30 (-3.84, 14.44)	6.83 (-2.53, 16.19)	6.15 (-0.32, 12.62)	5.31 (-1.51, 12.12)	5.15 (-1.74, 12.04)
FA.medium	-0.83 (-7.30, 5.64)	-2.71 (-9.46, 4.05)	-3.26 (-10.06, 3.54)	-2.47 (-7.81, 2.88)	-2.08 (-7.59, 3.43)	-1.83 (-7.43, 3.78)
FA.high	3.69 (-2.46, 9.84)	2.15 (-4.20, 8.50)	1.23 (-5.31, 7.76)	-2.52 (-8.43, 3.39)	-2.04 (-8.01, 3.93)	-1.82 (-7.88, 4.24)
UFA:SFA *B12	-1.72 (-7.96, 4.52)	-2.71 (-9.25, 3.83)	-3.14 (-9.70, 3.42)	0.10 (-6.76, 6.97)	-0.62 (-7.72, 6.49)	-0.58 (-7.79, 6.62)
UFA:SFA*FA.medium	1.59 (-4.51, 7.69)	2.60 (-3.67, 8.86)	2.19 (-4.11, 8.50)	0.99 (-4.61, 6.60)	0.74 (-4.88, 6.36)	0.30 (-5.50, 6.11)

UFA:SFA *FA.high	5.07 (-0.84, 10.98)	**6.44 (0.34, 12.53)**	**6.30 (0.16, 12.45)**	2.15 (-2.98, 7.27)	2.57 (-2.64, 7.78)	2.58 (-2.71, 7.86)
B12*FA.medium	-5.29 (-16.03, 5.46)	-8.07 (-19.15, 3.01)	-9.63 (-20.93, 1.67)	-7.96 (-15.72, -0.19)	-7.29 (-15.56, 0.99)	-6.80 (-15.20, 1.60)
B12*FA.high	-4.70 (-13.88, 4.48)	-7.62 (-17.13, 1.89)	-9.22 (-18.96, 0.52)	-0.82 (-9.20, 7.57)	-0.05 (-8.78, 8.69)	0.04 (-8.77, 8.85)
UFA:SFA*B12*FA.medium	0.65 (-6.71, 8.00)	1.82 (-5.96, 9.61)	2.35 (-5.48, 10.18)	0.01 (-8.02, 8.04)	1.42 (-6.76, 9.60)	1.25 (-7.03, 9.54)
UFA:SFA*B12*FA.high	0.35 (-6.17, 6.86)	1.40 (-5.47, 8.27)	1.68 (-5.21, 8.56)	0.26 (-7.23, 7.75)	0.81 (-6.97, 8.58)	0.86 (-7.02, 8.73)
